# Enhancing the Selective OH^−^ Adsorption for Durable Alkaline Seawater Oxidation at Industrial Current Densities

**DOI:** 10.1007/s40820-026-02133-8

**Published:** 2026-03-18

**Authors:** Shangshu Hu, Jiao Yang, Yujuan Zhuang, Xueyao Li, Han Xu, Fuwang Hu, Zhishuo Yan, Chao Liu, Jianmin Yu, Lishan Peng

**Affiliations:** 1 Key Laboratory of Rare Earths, Ganjiang Innovation Academy, Chinese Academy of Sciences, 341119 Ganzhou, People’s Republic of China; 2 School of Rare Earths, University of Science and Technology of China, 230026 Hefei, People’s Republic of China; 3 Institute of Applied Physics and Materials Engineering, University of Macau, 999078 Macao SAR, People’s Republic of China; 4 Jiangxi University of Science and Technology, 341000 Ganzhou, People’s Republic of China; 5 Department of Electrical and Computer Engineering, North Dakota State University, Fargo, 810052 USA

**Keywords:** Seawater electrolysis, NiFe-LDH, Heterostructure, Chloridion repulsion, Stability

## Abstract

The oxygen evolution reaction (OER) in seawater electrolysis is pivotal for sustainable hydrogen production, yet severe chloride ion (Cl^−^)-induced corrosion at the anode critically limits catalyst durability. Herein, we design a heterostructured catalyst comprising NiFe-layered double hydroxide and Ce(OH)CO_3_ (denoted as NiFe-LDH/Ce(OH)CO_3_) that exhibits remarkable OER stability in alkaline-simulated seawater. Experimental results and density functional theory calculations reveal that Ce(OH)CO_3_ incorporation modulates interfacial charge redistribution and enhances the Lewis acidity of Ni and Fe sites, thereby tuning the adsorption energetics of Cl^−^ and OH^−^. Time-of-flight secondary ion mass spectrometry further confirms the preferential adsorption of OH^−^ over Cl^−^, effectively suppressing Cl^−^-induced corrosion. As a result, NiFe-LDH/Ce(OH)CO_3_ demonstrates exceptional long-term stability, maintaining continuous operation for over 450 h at 1 A cm^−2^ in alkaline seawater. When integrated into an anion exchange membrane electrolyzer, the catalyst achieves 1 A cm^−2^ at a low cell voltage of 1.92 V and operates stably for over 60 h. The system delivers an impressive energy efficiency of 68.59% in alkaline-simulated seawater, corresponding to a hydrogen production cost as low as $0.97 per gasoline gallon equivalent at 500 mA cm^−2^.

**Graphical abstract:**

The integration of Ce(OH)CO_3_ with NiFe-LDH enhances the Lewis acidity of Ni and Fe, enabling preferential OH^-^ adsorption while eff ectively suppressing chlorine corrosion, and ultimately achieving durable seawater oxidation atindustrial current densities.

**Supplementary Information:**

The online version contains supplementary material available at 10.1007/s40820-026-02133-8.

## Highlights

- The introduced Ce(OH)CO_3_ optimizes charge distribution and enhances Lewis acidity of Ni/Fe sites, facilitating OH^−^ adsorption.
- The NiFe-layered double hydroxide/Ce(OH)CO_3_ enables stable alkaline seawater electrooxidation for over 450 h at a high current density of 1 A cm^−2^.
- In an anion exchange membrane system, an energy efficiency of 65.21% is attained at 1 A cm^−2^, with hydrogen production at a cost of USD 1.03 per gasoline gallon equivalent .

**Supplementary Information:**

The online version contains supplementary material available at 10.1007/s40820-026-02133-8.

## Graphical abstract

**Figure:**
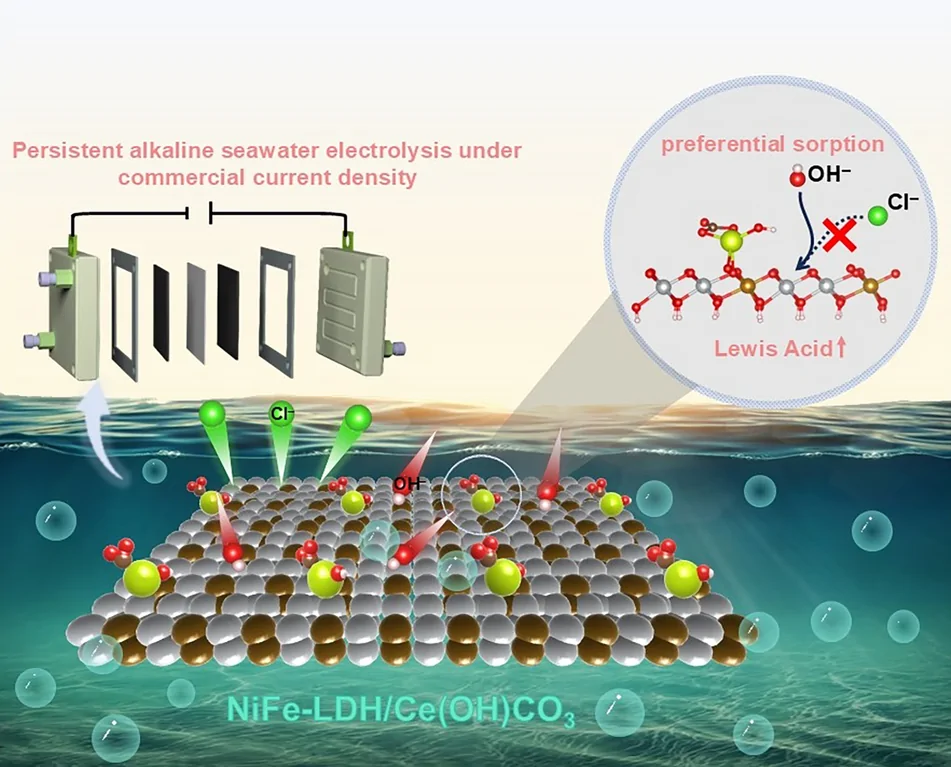

## Introduction

Hydrogen energy has emerged as a highly promising alternative to fossil fuels, owing to its exceptional energy density and environmentally benign, emission-free characteristics [1, 2]. The utilization of renewable energy sources to drive water electrolysis presents a sustainable, cost-effective, and eco-friendly pathway for generating storable hydrogen energy, addressing both energy security and environmental concerns [3, 4]. However, the extensive reliance on freshwater for electrolytic processes exacerbates global freshwater scarcity [5]. Given the abundant availability of seawater, its utilization for hydrogen production represents a promising long-term solution to address this challenge [6]. Nevertheless, the high concentration of Cl^−^ (approximately 0.5 M) in seawater not only competes with the OER at the anode during electrolysis but also induces severe corrosion of anode materials, posing significant technical hurdles [5, 7, 8]. The employment of precious metals, notably ruthenium oxide and iridium oxide, as anode materials is anticipated to address the issue of Cl^−^-induced corrosion on the anode surface in seawater electrooxidation (SWEO) process, attributable to their exceptional catalytic activity and stability [9]. Despite their advantages, the use of precious metal electrodes is constrained by their cost and scarcity. Consequently, developing efficient and affordable oxygen evolution reaction (OER) catalysts made from non-precious metals, such as transition metal oxides, chalcogenides, etc., is of utmost importance.

**Fig. 1:**
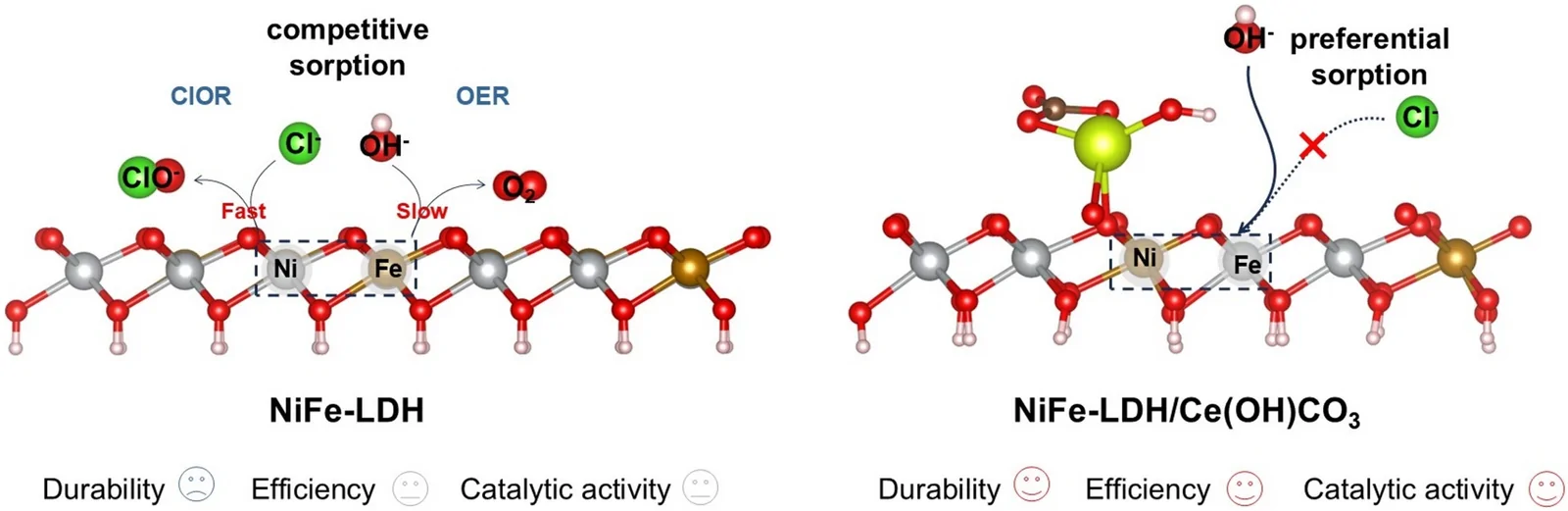
Schematic illustration of Cl^−^ corrosion in NiFe-LDH during seawater electrolysis (left) and the corrosion protection mechanism of NiFe-LDH/Ce(OH)CO_3_ (right)

Layered double hydroxide (LDH)-based anodes, such as NiFe-LDH, have garnered significant attention as non-precious metal catalysts, owing to their affordability, exceptional catalytic activity, and versatility in structure modulation [10]. However, during the process of alkaline seawater electrolysis, mitigating Cl^−^ corrosion of LDH-based anodes to ensure stable SWEO operation remains challenging [11]. Under harsh operational conditions (such as current densities exceeding 200 mA cm^−2^), the rapid generation of protons at active sites outstrips the supply of OH^−^, leading to local OH^−^ depletion and acidification. This acidic microenvironment enables concentrated Cl^−^ to compete effectively with the scarce OH^−^ for metal sites on the NiFe-LDH anode, initiating chloride oxidation reactions (ClOR) and consequent catalyst corrosion (Fig. 1) [12]. Current strategies to mitigate Cl^−^ corrosion in LDH-based anodes primarily rely on surface repulsion mechanisms, such as the introduction of Mo(VI) species into NiFe-LDH to enhance Cl^−^ repulsion through combined electrostatic and volume effects, thereby promoting stable seawater electrolysis [13–16]. Another effective strategy to mitigate Cl^−^ corrosion during SWEO involves constructing a protective barrier, as demonstrated by stabilizing LDH through an SO_4_^2−^ barrier formed by its preferential adsorption onto Ba^2+^-doped sites [17]. However, significant pH fluctuations under high current densities can nullify electrostatic repulsion, while intense ion flux and bubble evolution compromise physical barriers. Therefore, achieving durable seawater electrolysis at industrial-scale current densities remains a formidable challenge. To address this, we envisioned a paradigm shift from repelling Cl^−^ to selectively adsorbing OH^−^. According to the Pearson Hard-Soft Acid–Base (HSAB) principle, enhancing the Lewis acidity of the active sites in NiFe-LDH could preferentially favor OH^−^ adsorption over Cl^−^ [18]. Nevertheless, a systematic understanding of precisely tuning the Lewis acidity and its concomitant impact on the catalytic activity and corrosion resistance of NiFe-LDH anodes is still lacking. Moreover, bridging the gap between fundamental research and practical application necessitates the validation of these catalysts beyond conventional three-electrode cells, particularly in membrane electrode assemblies, such as anion exchange membrane electrolytic cell (AEMEC).

Herein, we employed a specific adsorption regulation strategy to boost the corrosion resistance of NiFe-LDH and achieved long-term alkaline SWEO at industrial current densities. The incorporation of Ce(OH)CO_3_ transforms the electron transfer pathway in NiFe-LDH from a Ni–O–Fe configuration to a Ce–O–Fe–O–Ni configuration. This rearrangement promotes electron transfer from Ni and Fe to Ce, thereby enhancing the Lewis acidity of both Ni and Fe. This enhancement promotes preferential OH^−^ adsorption over Cl^−^ on NiFe sites, effectively suppressing the chloride-induced corrosion pathway for robust SWEO (Fig. 1). In addition, the incorporation of Ce introduces abundant oxygen vacancies, significantly enhancing the electrical conductivity and intrinsic OER activity of the material. Density functional theory (DFT) calculations further revealed that the electron transfer from Ni/Fe to Ce sites establishes a thermodynamic preference for OH^−^ adsorption (ΔG_*OH_ = 0.67 eV) over Cl^−^ (ΔG_*Cl_ = 2.32 eV). The downshift of Ni/Fe d-band centers optimizes the adsorption strength of oxygen evolution intermediates (particularly *OOH), significantly reducing the energy barrier for the rate-determining step (RDS). The optimized catalyst exhibits outstanding SWEO performance, achieving a low overpotential of 221 mV at 100 mA cm^−2^ and remarkable stability over 450 h at industrial current density (1 A cm^−2^). When tested in an AEMEC, it also demonstrates stable operation for over 60 h at 500 mA cm^−2^. This work establishes a universal design strategy for developing durable LDH-based anodes toward practical SWEO applications.

## Experimental Section

### Materials

Nickel nitrate hexahydrate (Ni(NO_3_)_2_·6H_2_O), Hydrochloric acid (HCl), Sulfuric acid (H_2_SO_4_) and Anhydrous ethanol (C_2_H_5_OH) were acquired from the Xilong Science Co., Ltd., China. Ferric nitrate (Fe(NO_3_)_3_·9H_2_O), Ammonium chloride (NH_4_Cl), urea (CO(NH_2_)_2_), Potassium hydroxide (KOH), Deuterium oxide (D_2_O), Sodium thiosulfate (Na_2_S_2_O_3_), Potassium iodide (KI), Starch soluble (C_12_H_22_O_11_) and Sodium chloride (NaCl) were acquired from Macklin (Shanghai, China). Cerous nitrate (Ce(NO_3_)_3_·6H_2_O) was acquired from Aladdin (Shanghai, China). Nickel chloride (NiCl_2_) was acquired from Thermo Scientific (Beijing, China). Raney Ni was acquired from Baoshilai (Suzhou, China). All the chemicals were used directly without any further purification. Deionized (DI) water was used for catalyst preparation.

### Preparation of Coral-Like Nickel Foam, NiFe-LDH/Ce(OH)CO_3_ and RuO_2_

#### Preparations of the Coral-Like Nickel Foam

The coral-like nickel foam (NF) was prepared through a modified electrodeposition approach. Commercial NF was first ultrasonically cleaned in 3 M HCl for 20 min to eliminate surface NiO_*x*_ and impurities, then rinsed with deionized water and ethanol, and dried under vacuum. Electrodeposition was carried out using the cleaned NF (2 × 1.5 cm^2^) as the cathode and a ruthenium-iridium titanium plate as the anode in a solution of 2 M NH_4_Cl and 0.1 M NiCl_2_ at 2.5 A for 5 min. The resulting coral-like NF was treated with 0.1 M HCl, rinsed, and dried in N_2_.

#### ***Preparations of NiFe-LDH/Ce(OH)CO***_***3***_

For NiFe-LDH/Ce(OH)CO_3_ synthesis, a mixture of Ni(NO_3_)_2_·6H_2_O (2.64 mmol), Fe(NO_3_)_3_·9H_2_O (1.2 mmol), urea (16 mmol), and Ce(NO_3_)_3_·6H_2_O (0.96 mmol) in 30 mL deionized water was prepared. Four coral-like NF pieces were added, and the mixture was heated in a Teflon-lined autoclave at 150 °C for 12 h.

#### ***Preparations of RuO***_***2***_

For comparison, a commercial RuO_2_ catalyst ink was prepared by dispersing 5 mg of RuO_2_ powder in a solvent mixture containing 490 μL deionized water, 490 μL ethanol, and 20 μL of 5% Nafion solution. After 1h of ultrasonication, the homogeneous ink was drop-cast onto coral-like NF, yielding a uniform catalyst layer with a mass loading of 1 mg cm^–2^.

### Material Characterization

The crystal structure was analyzed using a D8-Advanced X-ray diffraction (XRD) with Cu K*α* radiation. Surface chemical states were examined by X-ray photoelectron spectroscopy (XPS, PHI Quantum 2000) with Al K*α* excitation (depth < 10 nm). Morphology and elemental distribution were studied using a Sigma 500 FE-SEM equipped with energy dispersive X-ray spectroscopy (EDS). The high-resolution transmission electron microscope (HRTEM) and selective area electron diffraction (SAED) were performed on a JEM-F200 microscope (200 kV, 0.19 nm resolution). Raman spectra were collected on an inVia reflex spectrometer with a 532 nm laser. Elemental composition was quantified by ICP-OES (PQ9000). XPS, SEM, and TEM were used to analyze post-stability samples of operation to assess structural and chemical evolution. The intensities of Cl^−^ and OH^−^ on the sample surface were measured using PHI nanoTOF Ⅱ Time-of-Flight SIMS (ULVAC-PHI.INC, JAPAN). Electron paramagnetic resonance (EPR) spectra were acquired using a Bruker EMX Plus-6/1 spectrometer. The in situ differential electrochemical mass spectrometry (DEMS) data were collected using a Shanghai Linglu QAS100 system. The X-ray absorption near edge structure (XANES) spectra are collected in TableXAFS-500A.

### Electrochemical Test

Electrochemical measurements were conducted using a CS2350M workstation (CORRTEST, Wuhan) with a standard three-electrode setup at room temperature, employing a carbon rod as the counter electrode and an Hg/HgO electrode as the reference. To ensure the accuracy of the potential readings, the saturated Hg/HgO reference electrode was first calibrated. All potentials recorded during the experiments were measured with respect to this calibrated electrode and were rigorously converted to the reversible hydrogen electrode (RHE) scale according to the theoretical equation. The potential was calibrated against the RHE following the equation:

1 $$ E_{{{\mathrm{RHE}}}} = E_{{{\mathrm{Hg}}/{\mathrm{HgO}}}} + \, 0.0{98 } + \, 0.0{59} \times {\mathrm{pH}} $$

Cyclic voltammetry (CV) was performed at a scan rate of 5 mV s^−1^. Electrochemical impedance spectroscopy (EIS) was conducted at an overpotential of 240 mV, covering a frequency range from 100,000 to 0.01 Hz, to analyze solution resistance (*R*_*s*_), redox transfer resistance (*R*_rt_), charge transfer resistance (*R*_ct_), and constant phase element (CPE). The OER performance was evaluated in 1 M KOH and 1 M KOH + 0.5 M NaCl. All potentials were converted to the reversible hydrogen electrode (RHE) scale and were IR-compensated. The compensation was applied at 90% according to the formula *E*_corr_ = *E* − iR_*s*_, where *E*_corr_ is the compensated potential, *E* is the measured potential, and *i* is the corresponding current. Stability was assessed via a constant-current test at 1 A cm^−2^, followed by CV analysis of the post-stability samples.

### Anion Exchange Membrane Electrolyzer

The anode of the AEMEC is a 1 cm × 1 cm NiFe-LDH/Ce(OH)CO_3_ catalyst, the cathode is a Raney Ni catalyst, and the anion exchange membrane uses PAP-TP-85 (40). The electrolyte consists of 1.0 M KOH and 0.5 M NaCl at a constant temperature of 80 °C.

### Theoretical Calculation Details

Spin-polarized DFT calculations were conducted on the projector-augmented wave (PAW) in the Vienna Ab initio Simulation Package (VASP) [19, 20]. The generalized gradient approximation (GGA) of Perdew–Burke–Ernzerhof (PBE) exchange functional was applied [21]. The cut-off energy for the plane-wave basis was set as 450 eV. A 20 Å vacuum slab in a direction perpendicular to the surface of the catalyst was adopted to avoid periodic interactions. The Brillouin zone integration was performed with 3 × 3 × 1 Monkhorst–Pack k-point sampling for geometry relaxation [22]. For the calculation of DOS, the k-point mesh was increased to 6 × 6 × 1. The convergence threshold for force and energy during optimization was set as 0.03 eV Å^−1^, and 10^−4^ eV, respectively. A 3 × 3 unit cell of NiFe-LDH surface was established. In the main text and supplementary information, the influence of neighboring intermediates on the energetics was not considered when free-binding energies were calculated without special instructions. Additionally, the influence of aqueous solvents in the calculation was not considered. The adsorbate intermediates were relaxed geometry optimization. On this basis, we simulated the adsorption behavior of *O, *OH, and *OOH intermediates for each catalyst, and each model was optimized for convergence. Δ*G* for each OER step was calculated through the model of computational hydrogen electrode along with the equation as follows [23]:

2 $$ \Delta G = \, \Delta E + \, \Delta {\mathrm{EZPE}} - T\Delta S $$

where Δ*E* refers to the DFT energy difference; Δ*S* and Δ*EZPE* refer to corrections with entropy through vibrational analysis and zero-point energy at 298.15 K, respectively.

## Results and Discussion

### Structural Characterizations

**Fig. 2:**
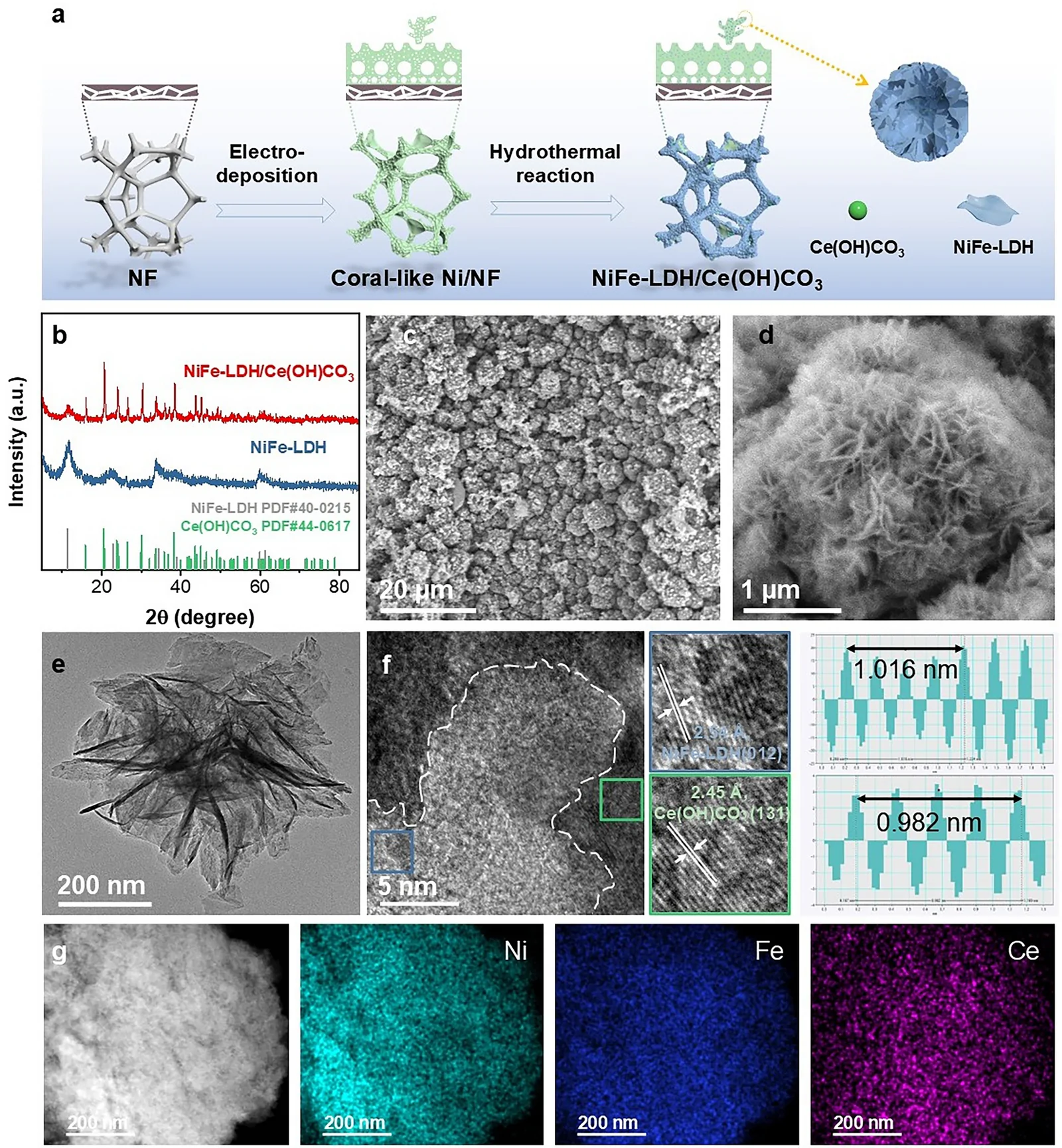
Structure description of NiFe-LDH and NiFe-LDH/Ce(OH)CO_3_. **a** Schematic illustration of NiFe-LDH/Ce(OH)CO_3_ preparation. **b** XRD patterns of NiFe-LDH and NiFe-LDH/Ce(OH)CO_3_. **c–e** SEM and TEM images of NiFe-LDH/Ce(OH)CO_3_. **f** HRTEM images of NiFe-LDH/Ce(OH)CO_3_. **g** HAADF image and EDS images of NiFe-LDH/Ce(OH)CO_3_

Figure 2a depicts the method for synthesizing NiFe-LDH/Ce(OH)CO_3_ in situ grown on NF. First, the commercial NF was electrodeposited in an ammonium chloride and nickel chloride solution, and the hydroelectricity bubbles formed in situ caused the porous nickel film of the electrodeposited product to adhere to the NF in the shape of coral [24]. Then, the coral-like NF was immersed in a solution containing metal salt precursors for a hydrothermal reaction to form heterostructured NiFe-LDH/Ce(OH)CO_3_ catalysts on the NF [25]. XRD patterns (Fig. 2b) exhibited that all samples possess diffraction peaks at 11.4°, 23.0°, and 34.43°, corresponding to the (003), (006), and (012) planes of NiFe-LDHs, respectively [26]. It is worth noting that the XRD patterns of NiFe-LDH/Ce(OH)CO_3_ contain diffraction peaks at 20.82°, 30.34°, and 38.51°, corresponding to the (020), (102), and (131) planes of Ce(OH)CO_3_, respectively, which confirms the coexistence of NiFe-LDH and Ce(OH)CO_3_ [27]. Within the composite’s pattern, the characteristic peaks of Ce(OH)CO_3_ are appreciably sharper compared to the neighboring LDH reflections, indicating a higher degree of structural order for the Ce(OH)CO_3_ phase in the hybrid material. Scanning electron microscopy (SEM) reveals that the NiFe-LDH/Ce(OH)CO_3_ nanoflowers are uniformly distributed on the surface of coral-like NF (Figs. 2c, d, and S1, S2). The nanoflower structure is conducive to exposing more active sites and gas release during electrooxidation. The TEM images further reveal that NiFe-LDH/Ce(OH)CO_3_ (Fig. 2e). The HRTEM image shows two distinct crystalline fringes: with spacings of 0.205 nm indexing to the (132) plane of Ce(OH)CO_3_ and 0.228 nm corresponding to the (015) plane of NiFe-LDH. Additionally, the SAED pattern confirms the presence of (143) and (012) crystallographic planes associated with Ce(OH)CO_3_ and NiFe-LDH, respectively (Figs. 2f, and S3–S5). The high-angle annular dark-field scanning transmission electron microscopy (HAADF-STEM) image and EDS mappings in Fig. 2g demonstrate an even distribution of Ni, Fe, and Ce elements across the NiFe-LDH/Ce(OH)CO_3_ nanosheets. Furthermore, the relative contents of Ni, Fe, and Ce are consistent with the expected composition (Fig. S6). These findings substantiate the successful synthesis of heterostructured NiFe-LDH/Ce(OH)CO_3_ on coral-like NF.

**Fig. 3:**
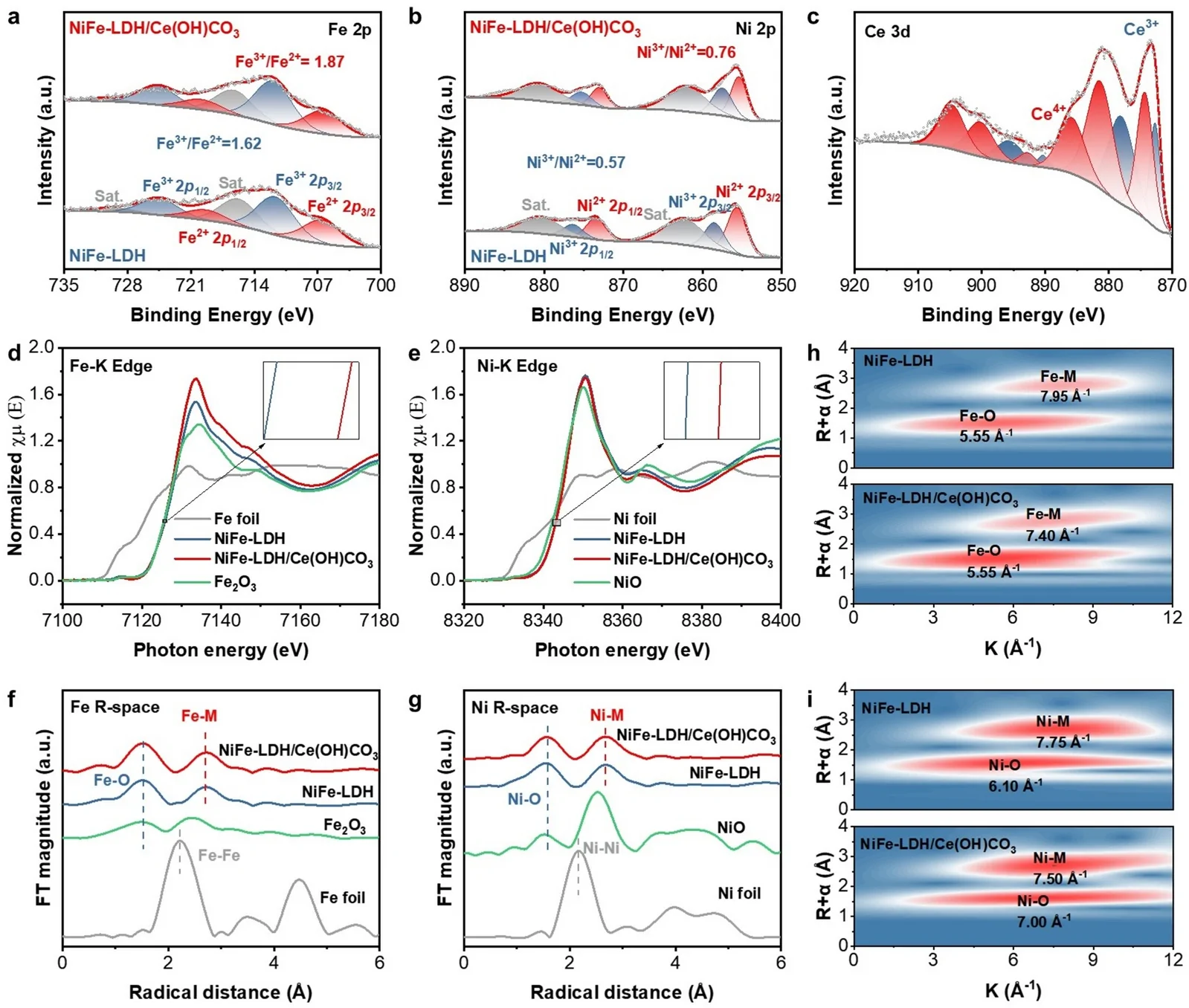
Chemical state and electronic structure of NiFe-LDH and NiFe-LDH/Ce(OH)CO_3_**. a** Fe 2*p*, **b** Ni 2*p*, and **c** Ce 3*d* XPS spectra of NiFe-LDH/Ce(OH)CO_3_ and NiFe-LDH. **d** Fe K-edge and **e** Ni K-edge XANES spectra of NiFe-LDH, NiFe-LDH/Ce(OH)CO_3_, Fe foil, and Fe_2_O_3_ reference samples. Fourier transforms of k^3^-weighted EXAFS spectra at **f** Fe and **g** Ni K-edges. Wavelet transforms of the k^3^-weighted EXAFS signals for **h** Fe K-edge and **i** Ni K-edge in NiFe-LDH and NiFe-LDH/Ce(OH)CO_3_

The surface structure and chemical states of NiFe-LDH and NiFe-LDH/Ce(OH)CO_3_ were characterized by Raman spectroscopy and XPS. The Raman spectra of both samples exhibited two primary bands positioned at 458 and 526 cm^−1^, which are attributable to the e_g_ bending and A_1*g*_ stretching vibrations of Ni–O in the Brucite-like LDH structure, respectively (Fig. S7). Additionally, two weaker bands are observed at 295 and 705 cm^−1^, linked to hydroxyl (O–H) groups [28]. A weak yet discernible band around 1047 cm^−1^, corresponding to the symmetric stretching vibration of carbonate anions (CO_3_^2−^) intercalated in the LDH structure, is present in both spectra [7, 29–32]. This confirms a consistent carbonate intercalation background across all samples in this study. The overall intensity of the peaks in NiFe-LDH/Ce(OH)CO_3_ is notably diminished, substantiating that a portion of NiFe-LDH on the surface of NiFe-LDH/Ce(OH)CO_3_ has been replaced by Ce(OH)CO_3_. The XPS survey spectrum of NiFe-LDH/Ce(OH)CO_3_ confirmed the presence of Ni, Fe, C, O, and Ce elements, with no detectable impurities (Fig. S8). The high-resolution XPS Fe 2*p* spectrum of NiFe-LDH exhibits the major peaks at 707.33 and 720.13 eV, which are assigned to Fe^2+^ 2*p*_1/2_ and 2*p*_3/2_, respectively, while the lateral peak corresponds to Fe^3+^ (711.60 and 724.40 eV) (Fig. 3a) [31]. By calculating the Fe^3+^/Fe^2+^ ratio based on peak area, a significant increase in Fe^3+^ content is observed in NiFe-LDH/Ce(OH)CO_3_ (1.87) compared with NiFe-LDH (1.62), due to the introduction of Ce species. The XPS Ni 2*p* spectrum of NiFe-LDH comprises characteristic peaks for Ni^2+^ (855.65 and 873.39 eV), as well as Ni^3+^ (858.48 and 876.33 eV) (Fig. 3b). The Ni^3+^/Ni^2+^ ratio in NiFe-LDH/Ce(OH)CO_3_ is calculated to be 0.76, which is higher than that in NiFe-LDH (0.57). These concomitant similar trends in Fe 2*p* and Ni 2*p* spectra indicate strong electronic interactions between NiFe-LDH and Ce(OH)CO_3_, likely arising from the establishment of an interface after Ce addition. Concurrently, the XPS Ce 3*d* spectrum can be deconvoluted into Ce^3+^ and Ce^4+^ components, confirming the successful incorporation of Ce species within the composite (Fig. 3c) [11, 33, 34]. The high proportion of Ce^4+^, acting as a strong Lewis acid, is expected to promote OER selectivity in seawater by facilitating OH⁻ adsorption. In the O 1*s* spectrum (Fig. S9) [35], three distinct peaks are resolved at 530.10, 531.35, and 533.41 eV, ascribed to oxygen vacancy (O_*v*_), hydroxyl oxygen (–OH), and surface-adsorbed water molecules (H_2_O_ads_), respectively. Compared to NiFe-LDH, an increased fraction of O_*v*_ and –OH ratio is observed for NiFe-LDH/Ce(OH)CO_3_, further supporting the electronic redistribution resulting from Ce introduction. The presence of these oxygen vacancies is independently confirmed by EPR spectroscopy (Fig. S10), which shows a discernibly enhanced characteristic signal at g ≈ 2.003 for the NiFe-LDH/Ce(OH)CO_3_ composite compared to the pristine NiFe-LDH. This signal is indicative of electrons trapped in oxygen vacancies and is consistent with the XPS findings [36]. Together, these results support the electronic redistribution resulting from Ce introduction. This enhancement of O_*v*_ mitigates Coulombic interactions between metal cations and oxygen anions, strengthens M–O covalency, and enables lattice oxygen involvement in surface redox reactions for OER.

To probe the electronic states and local coordination environments of Ni and Fe in NiFe-LDH/Ce(OH)CO_3_, we conducted XANES and extended X-ray absorption fine structure (EXAFS) analyses. The Fe K-edge XANES spectra (Fig. 3d) reveal a positive shift for NiFe-LDH/Ce(OH)CO_3_ compared to NiFe-LDH. Quantitative valence analysis using the integration method indicates an increase in the Fe oxidation state from 2.95 in NiFe-LDH to 3.00 in NiFe-LDH/Ce(OH)CO_3_ (Fig. S11). Similarly, the Ni K-edge spectra show absorption edges above that of NiO for both samples, with the Ni valence state increasing markedly from 2.52 in NiFe-LDH to 3.01 in NiFe-LDH/Ce(OH)CO_3_ (Fig. 3e). These results demonstrate that the introduction of Ce(OH)CO_3_ concurrently elevates the oxidation states of both Fe and Ni in NiFe-LDH, which is consistent with the XPS findings. Extended X-ray absorption fine structure (EXAFS) analysis of the Fe K-edge reveals similar structural features between NiFe-LDH/Ce(OH)CO_3_ and NiFe-LDH. The intense peaks at 1.50 and 2.71 Å correspond to Fe–O and Fe–Ni/Fe (Figs. 3f, and S12, S13). Analogous characteristics are observed in the Ni K-edge spectra, with intense peaks at 1.58 Å (Ni–O) and 2.68 Å (Ni–Fe/Ni) (Fig. 3g). Building on this, further EXAFS analysis provides detailed insights into the coordination changes induced by the introduction of Ce(OH)CO_3_ (Fig. S14). At the Fe K-edge, the heterostructure exhibits an increased relative intensity of the Fe–O scattering path at approximately 1.5 Å compared to pristine NiFe-LDH, indicating a strengthened Fe–O coordination that is attributable to bonding with oxygen species from Ce(OH)CO_3_. Moreover, the Fe–M (M = Ni/Ce) shell, located near 2.71 Å, exhibits a positive shift and broadening, strongly suggesting the formation of an interfacial Fe–O–Ce bond and confirming substantial interaction at the heterojunction [37, 38]. In contrast, the Ni K-edge spectrum reveals minimal change in the first-shell Ni–O coordination. To quantify these observed changes, further EXAFS fitting (Tables S1 and S2) reveals that the introduction of Ce(OH)CO_3_ increases the coordination numbers of both Fe–O (from 5.80 to 6.17) and Ni–O (from 6.34 to 6.84). This coordination evolution corroborates the elevated oxidation states of Ni and Fe, which enhance their Lewis acidity. Integrating the evidence from both absorption edges, it is proposed that Ce(OH)CO_3_ promotes the establishment of a coherent interfacial Ni–O–Fe–O–Ce atomic arrangement, elucidating the structural origin of the enhanced catalytic properties. Moreover, wavelet transform analysis reveals a distinct distortion in the local coordination geometry, characterized by a shift in the metal centers (Fig. 3h, i). This can be attributed to the formation of interfacial Ni–O–Fe–O–Ce bonds [37]. Collectively, these findings provide direct evidence for strong interfacial interactions within the NiFe-LDH/Ce(OH)CO_3_ heterostructure. The enhanced interfacial coupling is pivotal in optimizing the preferential adsorption of OH^−^ over Cl^−^ during catalysis.

### Electrocatalytic Measurement

**Fig. 4:**
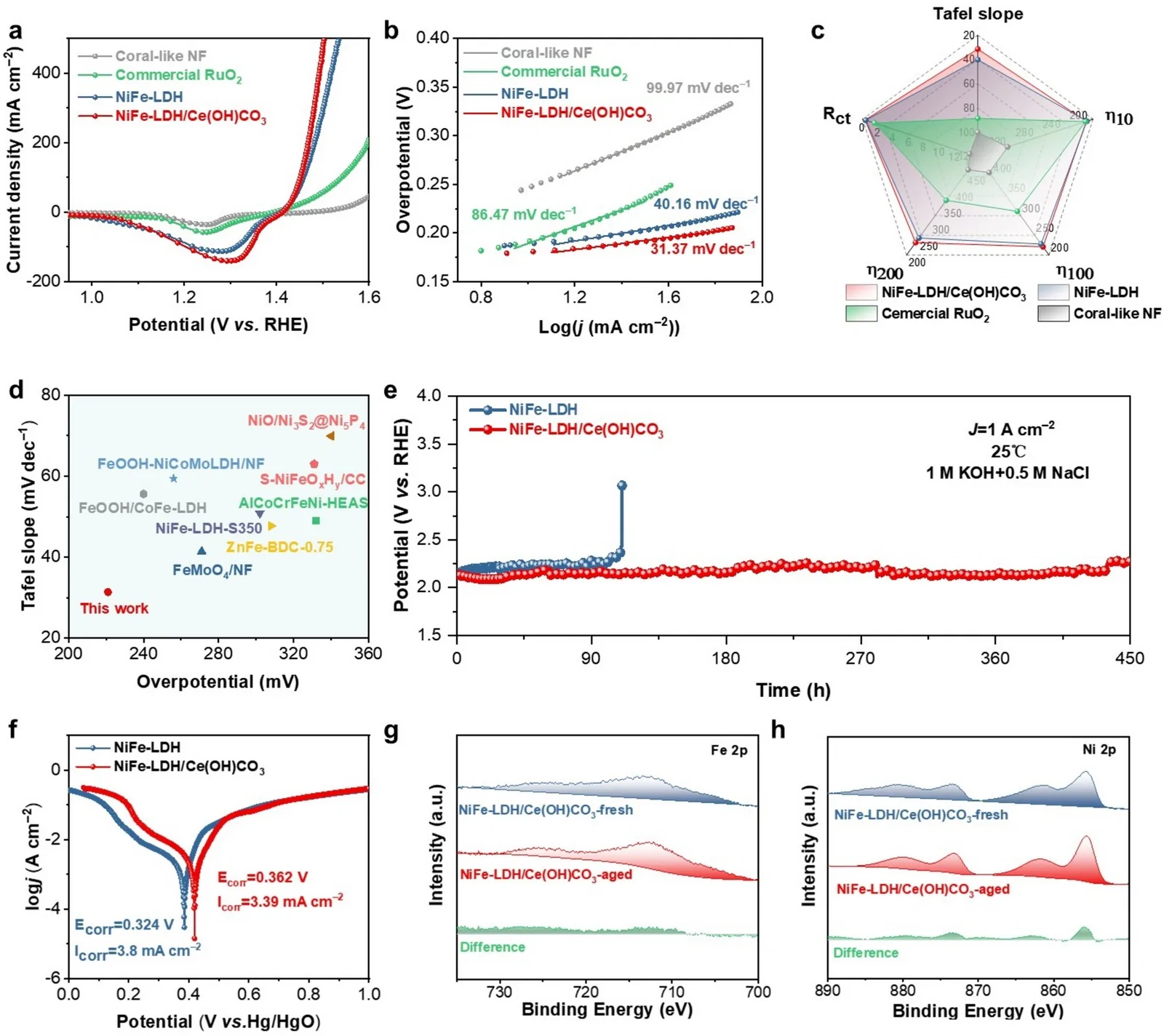
OER activity of coral-like NF, commercial RuO_2_, NiFe-LDH and NiFe-LDH/Ce(OH)CO_3_ and SWEO stability test of NiFe-LDH and NiFe-LDH/Ce(OH)CO_3_. **a** LSV polarization curves. **b** Tafel plots. **c** Multivariate radar chart. **d** Comparisons of state-of-the-art works of the OER activities with this work. **e** Chronopotentiometry curve. **f** Corrosion polarization plots. High-resolution XPS spectra: **g** Fe 2*p* and **h** Ni 2*p* of NiFe-LDH/Ce(OH)CO_3_

OER measurements were examined in simulated seawater samples (SSW) composed of 1 M KOH and 0.5 M NaCl to illustrate the electrochemical characteristics of NiFe-LDH/Ce(OH)CO_3_ and these reference samples (Fig. S15 and Table S3) [39, 40]. The polarization curve of NiFe-LDH/Ce(OH)CO_3_ shows a lower onset potential (1.42 V), demonstrating superior electrochemical performance characteristics (Fig. 4a). NiFe-LDH/Ce(OH)CO_3_ exhibits a Tafel slope of 31.37 mV dec^−1^ (Fig. 4b), which is significantly lower than that of NiFe-LDH (40.16 mV dec^−1^), commercial RuO_2_ (86.47 mV dec^−1^), and coral-like NF (99.97 mV dec^−1^). In addition, the NiFe-LDH/Ce(OH)CO_3_ requires an overpotential of 221 mV to reach a current density of 100 mA cm^−2^, outperforming NiFe-LDH (228 mV), commercial RuO_2_ (311 mV) and coral-like NF (410 mV) (Fig. S16). The optimal overpotential and Tafel slope of NiFe-LDH/Ce(OH)CO_3_ confirm its best OER activity and fastest catalytic kinetics. The smallest reaction charge transfer resistance (*R*_ct_) of NiFe-LDH/Ce(OH)CO_3_ further demonstrates its superior OER performance (Fig. S17 and Table S4). The reason for this phenomenon may be that the introduction of Ce(OH)CO_3_ nanosheets can optimize the electron-filling state and accelerate the charge transfer between the catalytic site and the oxy-containing adsorption, thus improving the effect of OER [34]. The multivariate radar chart (Fig. 4c) quantitatively compares the performance of NiFe-LDH/Ce(OH)CO_3_ against several other materials across key metrics. NiFe-LDH/Ce(OH)CO_3_ demonstrates comprehensive enhancements, including lower overpotential, reduced charge transfer resistance (*R*_ct_), and a decreased Tafel slope. The analysis of the electrochemically active surface area (ECSA) by double-layer capacitance (*C*_dl_) is one of the means to understand the principles of OER performance improvement (Fig. S18) [41, 42]. As shown in Fig. S19, NiFe-LDH/Ce(OH)CO_3_ exhibits a larger *C*_dl_ of 14.43 mF cm^−2^, surpassing that of NiFe-LDH (12.38 mF cm^−2^). This enhancement is attributed to Ce(OH)CO_3_ nanosheets intercalating between LDH nanoflowers, reducing their aggregation while maintaining active site accessibility, significantly contributing to the improved OER performance [43]. The CV profiles of NiFe-LDH/Ce(OH)CO_3_ and NiFe-LDH exhibit similar oxidation–reduction features, with oxidation peaks corresponding to NiFeOOH formation during the anodic sweep and reduction peaks during the cathodic sweep [30]. However, NiFe-LDH/Ce(OH)CO_3_ shows a lower oxidation potential and a more prominent reduction peak, suggesting that Ce(OH)CO_3_ enhances the redox activity of Ni and Fe species, further boosting OER efficiency (Fig. S20). In comparison with most reported OER catalysts, the NiFe-LDH/Ce(OH)CO_3_ catalyst demonstrates superior OER performance during the seawater electrolysis process (Fig. 4d and Table S5). The electrocatalytic performance of the samples was then evaluated in the pretreated, alkaline real seawater (Fig. S21). As derived from the polarization curves, the NiFe-LDH/Ce(OH)CO_3_ catalyst achieved a current density of 100 mA cm^−2^ at an overpotential of 350 mV. This performance is superior to that of the control samples: NiFe-LDH (433 mV), commercial RuO_2_ (485 mV), and coral-like NF (506 mV) required significantly higher overpotentials to reach the same current density. Further kinetics analysis revealed a Tafel slope of 91.71 mV dec^−1^ for the NiFe-LDH/Ce(OH)CO_3_ catalyst, which is considerably lower than those of NiFe-LDH (96.31 mV dec^−1^), commercial RuO_2_ (131.33 mV dec^–1^, and coral-like NF (143.03 mV dec^−1^). We note that all catalysts exhibited a degree of performance degradation in real seawater compared to simulated electrolyte, due to the inherent complexity and interfering components present in natural seawater [44]. Nevertheless, the NiFe-LDH/Ce(OH)CO_3_ maintained its relative performance advantage. To gain a fundamental understanding of the OER mechanism on NiFe-LDH/Ce(OH)CO_3_, we conducted DEMS coupled with ^18^O isotopic labeling (Fig. S22). The calculated ^34^O_2_/^32^O_2_ ratio on NiFe-LDH/Ce(OH)CO_3_ is approximately 0.83%, which indicates that the OER primarily proceeds via the adsorbate evolution mechanism (AEM) with minor involvement of the lattice oxygen mechanism (LOM) [45]. This finding corresponds to the trace amount of oxygen vacancies identified in our previous XPS analysis. Collectively, the DEMS results provide direct evidence that the OER on NiFe-LDH/Ce(OH)CO_3_ favors the AEM pathway.

The stability of electrocatalysts is a crucial parameter in evaluating their effectiveness for SWEO [46]. In this study, we subjected NiFe-LDH and NiFe-LDH/Ce(OH)CO_3_ to constant-current testing at an industrial current density of 1 A cm^−2^ in SSW. The NiFe-LDH/Ce(OH)CO_3_ catalyst maintains its performance for over 450 h, significantly outperforming the NiFe-LDH catalyst, which degrades sharply after only 110h (Fig. 4e). Moreover, this stability surpasses that of most recently reported anode catalysts under similar alkaline seawater electrolysis conditions (Fig. S23 and Table S6). This result underscores the superior operational lifetime of NiFe-LDH/Ce(OH)CO_3_ for SWEO. CV analysis demonstrates only a negligible decline in electrochemical activity, further validating the exceptional stability of the NiFe-LDH/Ce(OH)CO_3_ under industrial current densities for SWEO (Fig. S24). Beyond stability, anode selectivity is equally critical for practical seawater electrolysis. The OER selectivity and corresponding Faradaic efficiency (FE) were evaluated in an H-type cell using NiFe-LDH/Ce(OH)CO_3_ as the anode and commercial Raney nickel as the cathode. Chloride oxidation activity was monitored by iodometric titration of the electrolyte (Figs. S25–S27). In an alkaline-simulated seawater environment, the OER FE reached ~ 98%, whereas the FE for hypochlorite (ClO^−^) formation remained as low as ~ 0.5%. The slight deviation of the total FE from 100% falls within the intrinsic measurement uncertainty of the gasometric method. These results highlight the outstanding OER selectivity of NiFe-LDH/Ce(OH)CO_3_, establishing it as a highly promising anode material for alkaline seawater splitting [45, 47]. The exceptional performance can be attributed to the composite’s enhanced structural integrity and corrosion resistance. Electrochemical corrosion tests revealed that the NiFe-LDH/Ce(OH)CO_3_ exhibits a more positive corrosion potential and a lower corrosion current density than NiFe-LDH, thereby confirming its superior corrosion resistance properties (Fig. 4f). SEM and TEM analyses demonstrated that the nanostructure of the NiFe-LDH/Ce(OH)CO_3_ exhibits no significant alterations after the stability tests (Figs. S28 and S29). HRTEM analysis identifies distinct lattice fringes corresponding to NiFe-LDH and Ce(OH)CO_3_ phases, along with amorphous regions potentially forming through oxidative processes during OER (Fig. S30). XRD patterns further confirm the structural stability, showing no emergence of new diffraction peaks and only a slight negative shift in all peaks, which can be attributed to minor lattice expansion during prolonged operation (Fig. S31). Furthermore, EDS mappings demonstrated that Ni, Fe, and Ce are still evenly distributed (Figs. S32 and S33). Comprehensive spectroscopic characterization through XPS and Raman spectroscopy revealed that the NiFe-LDH/Ce(OH)CO_3_ composites maintained their intrinsic chemical composition, surface morphology, and bond configuration, with only a marginal increase in metal valence, consistent with the electrooxidation process following prolonged constant-current seawater electrooxidation testing (Figs. 4g, h and S34–S36). The leaching of Ni and Fe into the electrolyte after stability testing was further quantified by ICP analysis (Fig. S37). The results show that the amounts of Ni (0.01 mg L^−1^) and Fe (0.14 mg L^−1^) leached from NiFe-LDH/Ce(OH)CO_3_ are markedly lower than those from the pristine NiFe-LDH (Ni: 0.73 mg L^−1^; Fe: 0.32 mg L^−1^). These phenomena indicate that NiFe-LDH/Ce(OH)CO_3_ possesses good structural stability in the SWEO process.

### Mechanism Investigation

**Fig. 5:**
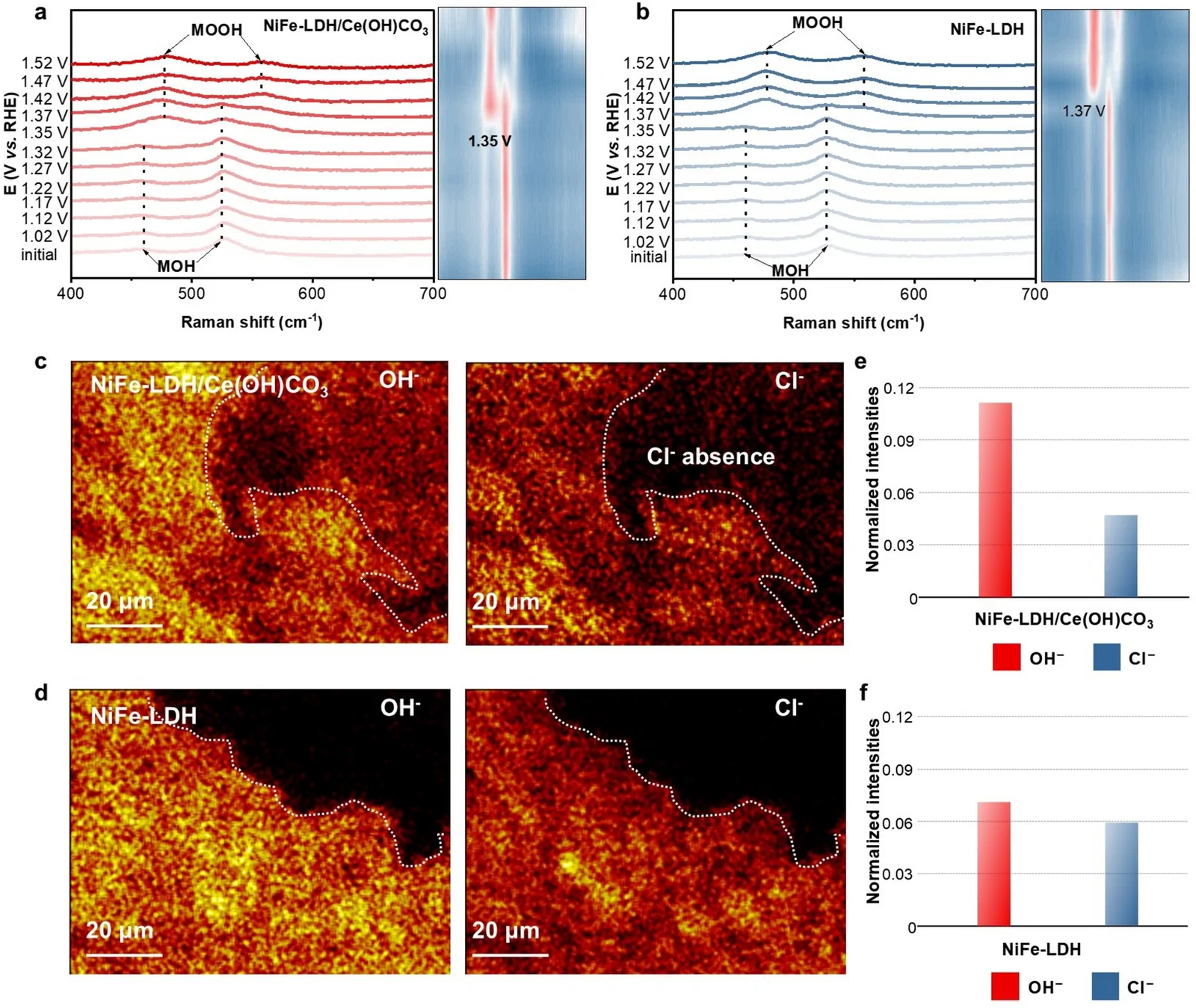
Operando Raman spectra of **a** NiFe-LDH/Ce(OH)CO_3_ and **b** NiFe-LDH. TOF–SIMS mapping for **c** NiFe-LDH/Ce(OH)CO_3_ and **d** NiFe-LDH after the stability test; **e, f** corresponding OH^−^/Cl^−^ intensity ratios

To explore the OER catalytic behavior of NiFe-LDH/Ce(OH)CO_3_ in SSW, in situ Raman spectroscopic was performed within a potential window of 0.92–1.52 V (vs. RHE) (Fig. 5a, b). The characteristic bands at 458 and 526 cm^−1^ detected on both electrodes can be assigned to the e_*g*_ bending mode and A_1*g*_ stretching modes of metal hydroxide (M–OH, M = Fe, Ni) species, respectively. When the anodic potential increases to 1.37 V (vs. RHE), the pristine NiFe-LDH electrode exhibits a pair of new peaks at 478 and 560 cm^−1^, which are attributed to the vibrational modes characteristic of metal oxyhydroxide (M–OOH) formation. This spectroscopic evolution unambiguously evidences the phase transition from *α*-NiFe-LDH to *γ*-NiFeOOH, accompanied by the oxidation of Ni^2+^ to higher valence states (Ni^3+^/Ni^4+^). In comparison, the NiFe-LDH/Ce(OH)CO_3_ electrode triggers the *α*/*γ* transformation at a lower overpotential (1.35 V vs. RHE), in agreement with the increased oxidation peak area observed in the linear sweep voltammetry (LSV) measurements. This result suggests that the addition of Ce(OH)CO_3_ promotes Ni oxidation, endowing the material with superior pre-catalytic activation (i.e., structural pre-reconstruction) capability [16]. Moreover, the calculated band intensity ratios (I_560_/I_478_) of NiFe-LDH/Ce(OH)CO_3_ are higher than those of the pure NiFe-LDH, indicating an increased structural disorder within the NiOOH phase, which correlates with the substantially improved oxygen evolution activity (Fig. S38) [12].

We then investigate the state and functional role of Ce(OH)CO_3_ in enhancing operational stability. Time-of-flight secondary ion mass spectrometry (TOF–SIMS) was employed to quantify the surface concentrations of OH^−^ and Cl^−^ on the activated electrode. As shown in Fig. 5c, the TOF–SIMS mapping of NiFe-LDH/Ce(OH)CO_3_ indicates a surface predominantly enriched with OH^−^. In contrast, the Cl^−^ signal is extremely weak, even falling below the detection limit within the dashed region. In contrast, the NiFe-LDH electrode exhibits co-localized distributions of OH^−^ and Cl^−^, demonstrating limited Cl⁻ repulsion (Fig. 5d). Analysis of the OH^−^/Cl^−^ signal intensity ratio further confirms that the NiFe-LDH/Ce(OH)CO_3_ electrode facilitates notable enrichment of OH^−^ while suppressing the adsorption of Cl^−^ on the metal active sites [48], exhibiting a ratio of 2.38 compared to 1.20 for pristine NiFe-LDH (Fig. 5e, f). These results clearly demonstrate that the incorporation of Ce(OH)CO_3_ selectively promotes OH^−^ adsorption and effectively inhibits Cl^−^ binding, thereby contributing to improved OER selectivity and operational stability in seawater electrolysis.

**Fig. 6:**
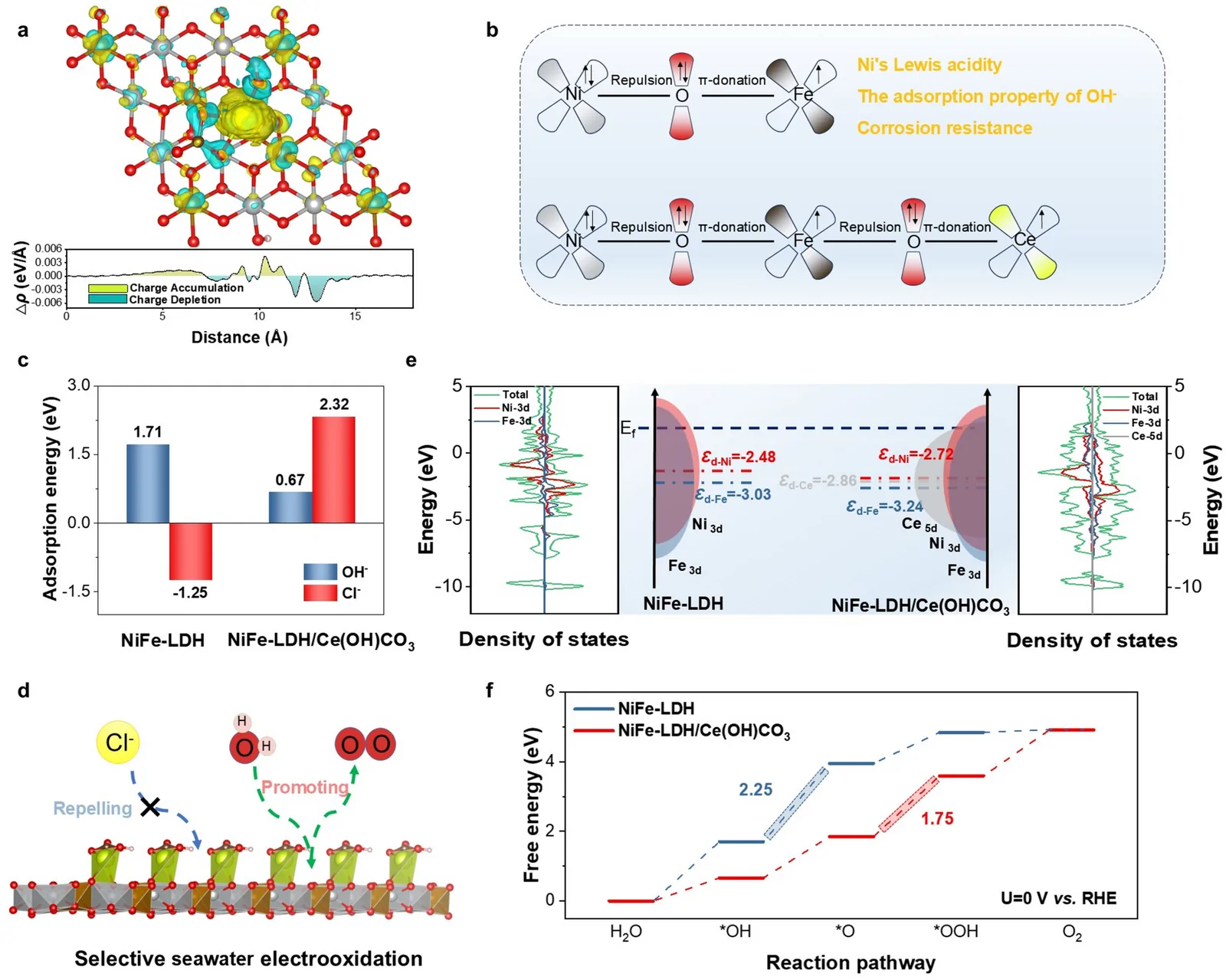
**a** Side view of the differential charge density for NiFe-LDH/Ce(OH)CO_3_. The yellow-green regions (positive isosurfaces) represent electron accumulation, while cyan regions (negative isosurfaces) indicate electron depletion. **b** Schematics of the electronic interplay among Ni, Fe, Ce, and O in NiFe-LDH/Ce(OH)CO_3_. **c** Adsorption energies of OH^−^ and Cl^−^ on NiFe-LDH and NiFe-LDH/Ce(OH)CO_3_ surface. **d** Schematic illustration of the SWEO mechanism on NiFe-LDH/Ce(OH)CO_3_. **e** Projected density of states (PDOS) and schematic band structure diagrams of NiFe-LDH and NiFe-LDH/Ce(OH)CO_3_. **f** Comparison of OER energy profiles on NiFe-LDH and NiFe-LDH/Ce(OH)CO_3_ surface

To elucidate the origin of the enhanced OER activity and stability, DFT calculations were conducted on NiFe-LDH and its Ce(OH)CO_3_-modified counterpart. Guided by XAFS analysis, representative structural models were constructed and optimized to capture the key atomic features of both systems (Fig. S39). Differential charge density (Δ*ρ*) analysis reveals the electron accumulation (Δ*ρ* > 0) around Ce and partial depletion (Δ*ρ* < 0) around Ni/Fe at the NiFe-LDH/Ce(OH)CO_3_ interface, indicating that the incorporation of Ce(OH)CO_3_ induces interfacial charge redistribution (Fig. 6a). This is further quantified by Bader charge analysis, which shows that the charge on Ni increases from − 1.28 to − 1.14 and on Fe from − 1.60 to − 1.56 upon Ce(OH)CO_3_ incorporation (Fig. S40). The consistent decrease in electron density around Ni and Fe unequivocally confirms electron transfer from Ni/Fe to Ce. Charge-transfer processes can rationalize this behavior through the Ni–O–Fe unit, where the strong electron–electron repulsion between Ni^2+^(t_2*g*_^6^e_*g*_^2^) and O^2−^, together with weak *π*-donation from Fe^3+^ (t_2*g*_^5^e_*g*_^0^) via the Fe–O bridge, mediates electron delocalization (Fig. 6b). As a result of Ce modification, an extended Ni–O–Fe–O–Ce bridging framework is formed. The strongly electron-deficient *d*-orbitals of Ce^3+^ drive partial electron transfer from Ni and Fe to Ce via the bridging oxygen atoms, indicating a strong electronic coupling characterized by the specific and directional reconstruction of the electronic structure at the active sites, beyond a simple charge depletion. This thereby depletes electron density at Ni and Fe sites and enhancing their Lewis acidity, as further evidenced by Crystal Orbital Hamiltonian Population (COHP) analysis. This analysis reveals a significantly weakened bonding interaction for the competing Cl^−^ adsorbate on the composite, directly demonstrating that the electronic coupling differentially modulates the frontier orbital characteristics (Fig. S41) [49]. The enhanced Lewis acidity is thus corroborated by XPS and XAFS analysis. According to the HSAB principle, the highly oxidized Ni and Fe sites serve as strong Lewis acids, where the tailored electronic structure through coupling fosters a high and selective affinity for OH^−^ over other anions like Cl^−^. This selective adsorption not only enhances the catalytic selectivity for the OER but also effectively suppresses metal leaching [50]. As depicted in Fig. 6c, Ce(OH)CO_3_ alters the adsorption behavior of NiFe-LDH by lowering the OH⁻ adsorption energy from 1.71 to 0.67 eV while increasing the Cl^−^ adsorption energy from − 1.25 to 2.32 eV. Meantime, the OH^−^ adsorption is more favorable than Cl^−^ adsorption in the NiFe-LDH/Ce(OH)CO_3_ system (0.67 vs. 2.32 eV), reflecting that OH^−^ is more likely to occupy the active sites. This result suggests that Cl^−^ binding to the metal sites is effectively hindered, thereby ensuring stable seawater electrolysis (Fig. 6d). Additionally, the presence of Ce(OH)CO_3_ can influence the electronic structure in NiFe-LDH, as evidenced by the projected density of states (PDOS). With the addition of Ce(OH)CO_3_, the d-band centers of Ni and Fe undergo a downward shift of 0.285 eV (from − 2.476 to − 2.761 eV) and 0.213 eV (from − 3.028 to − 3.241 eV) relative to the Fermi level, respectively (Fig. 6e). This negative shift reflects the electron delocalization effect induced by Ce, which adjusts the interaction strength between the metal active center and the reactants by lowering the energy levels of unoccupied antibonding states. Gibbs free energy analysis further reveals that Ce(OH)CO_3_ integration significantly alters the OER thermodynamics (Fig. 6f). Comparative assessment of rate-determining steps (RDS) shows that the pristine NiFe-LDH exhibits an energy barrier of 2.247 eV for the *OH to *O conversion, whereas the Ce-modified system presents a reduced barrier of 1.748 eV for the subsequent *O to *OOH step, corresponding to a 22.3% decrease in activation energy. These results indicate that the introduction of Ce(OH)CO_3_ effectively facilitates OER kinetics and enhances overall catalytic activity.

### Catalytic Performance in Seawater AEMEC

**Fig. 7:**
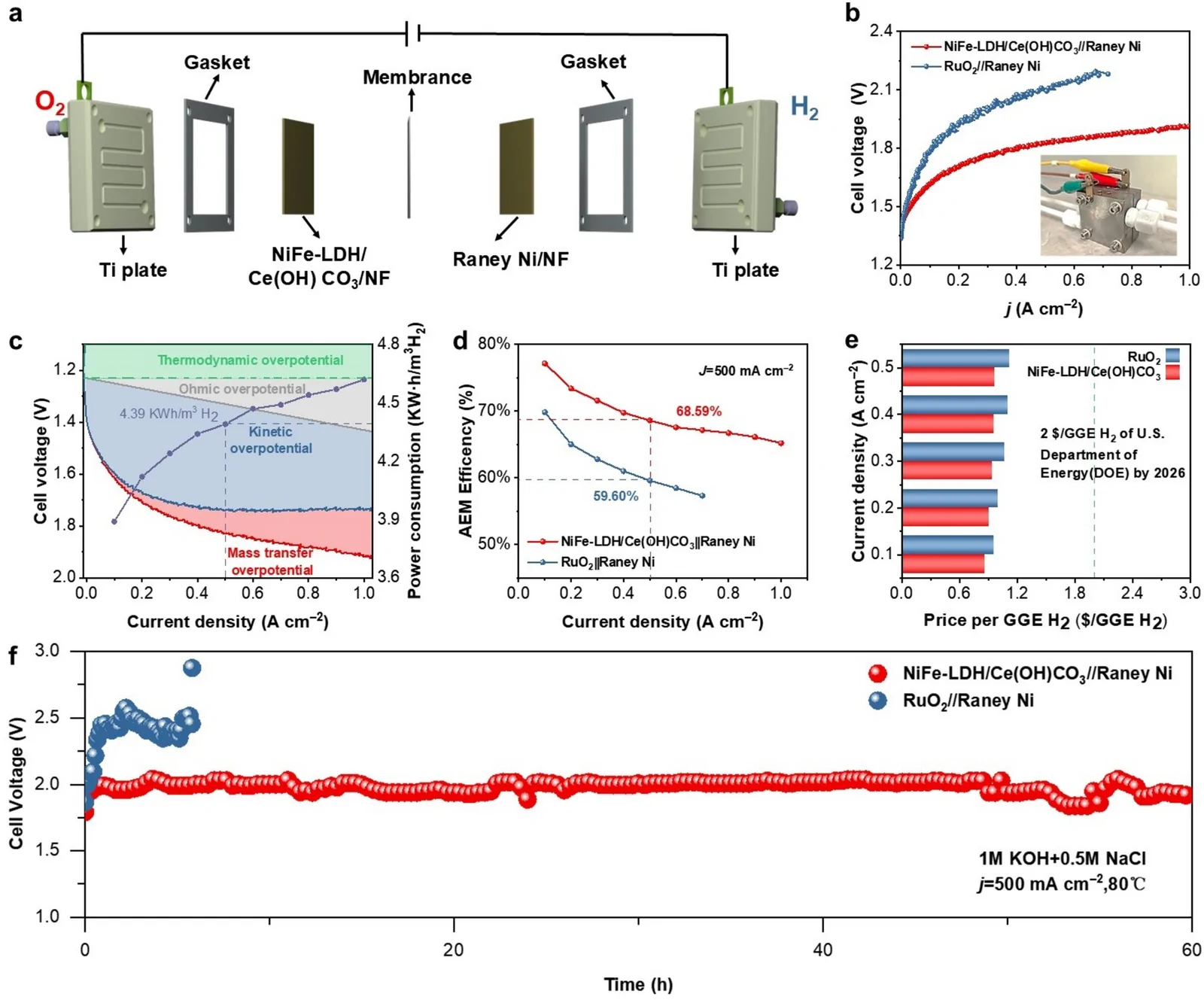
**a** Diagram of an AEMEC. **b** Overall seawater splitting performance of NiFe-LDH/Ce(OH)CO_3_/NF||Raney Ni/NF and RuO_2_/NF||Raney Ni/NF. All potentials were iR-corrected using solution resistances of 0.62 and 0.49 Ω, respectively, with a compensation level of 90%. **c** Power consumption, **d** efficiency, and **e** estimated cost for producing 1.0 kg of hydrogen by the AEMEC. All measurements were performed in 1.0 M KOH + 0.5 M NaCl across a range of current densities. **f** Chronopotentiometry curve of NiFe–LDH/Ce(OH)CO_3_/NF||Raney Ni/NF and RuO_2_/NF||Raney Ni/NF pair in 1 M KOH + 0.5 M NaCl electrolyte

To assess the potential of NiFe-LDH/Ce(OH)CO_3_ for industrial application in seawater splitting, we assembled a flow cell reactor with a membrane electrode assembly (MEA) incorporating 1 cm × 1 cm electrodes (Fig. 7a). The anode catalyst was NiFe-LDH/Ce(OH)CO_3_/NF (or, for comparison, RuO_2_/NF), while the cathode was commercial Raney Ni for overall seawater electrolysis. The density functional theory curve recorded in a 1 M KOH solution with the addition of 0.5 M NaCl demonstrates that the NiFe-LDH/Ce(OH)CO_3_/NF||Raney Ni/NF electrode achieves a current density of 1 A cm^−2^ at 1.92 V, significantly surpassing the performance of the RuO_2_/NF||Raney Ni/NF catalyst, which reaches only 0.30 A cm^−2^ at 2 V (Fig. 7b). The energy consumption and operational efficiency of the AEMEC were further evaluated in 1.0 M KOH + 0.5 M NaCl at various current densities (Fig. 7c, d; Tables S7 and S8). The system exhibited efficiencies of 77.16% and 68.59% at cell voltages of 1.62 and 1.83 V, corresponding to current densities of 100 and 500 mA cm^−2^, respectively. Notably, the estimated hydrogen production cost reached as low as $0.97 per GGE of H_2_ (Fig. 7e), which is below the US DOE’s 2026 target of $2.00 per GGE. Furthermore, the electrode demonstrated excellent stability, maintaining continuous operation for over 60 h at 500 mA cm^−2^ (Fig. 7f). These results indicate that the NiFe-LDH/Ce(OH)CO_3_ catalyst possesses high activity, durability, and economic feasibility under industrially relevant conditions, underscoring its promise for large-scale hydrogen production via seawater electrolysis.

## Conclusions

In summary, we have designed a corrosion-resistant and efficient catalyst by integrating Ce(OH)CO_3_ with NiFe-LDH for durable seawater oxidation at industrial-level current densities. The incorporation of Ce(OH)CO_3_ forms a Ce–O–Fe–O–Ni catalytic unit at the NiFe-LDH interface. This unit redistributes electrons from Ni/Fe to Ce, inducing high-valent Ni and Fe species with enhanced Lewis acidity, which promotes preferential OH^−^ adsorption over Cl^−^ and effectively suppresses chloride-induced corrosion, as confirmed by TOF–SIMS and DFT calculations. In addition, this modification downshifts the Ni/Fe d-band centers and lowers the energy barrier of the RDS, accelerating OER kinetics. As a result, the obtained NiFe-LDH/Ce(OH)CO_3_ catalyst exhibits both superior catalytic activity and outstanding stability, delivering a low overpotential of 221 mV at 100 mA cm^−2^ and continuously operating at a high current density of 1 A cm^−2^ for over 450 h without significant decay. Moreover, seawater AEMEC with industrial current density (500 mA cm^−2^) and durability (60 h) is achieved by using a NiFe-LDH/Ce(OH)CO_3_ anode and a Raney Ni cathode, with an electrolysis efficiency of 68.59% and an energy consumption of 4.39 kWh kg^−1^ H_2_. This work provides a practical and general strategy for developing durable LDH-based anodes for seawater electrolysis.

## Supplementary Information

Below is the link to the electronic supplementary material.

Supplementary file1 (DOCX 7073 KB)
