# Supplementary material for: Enhancing the Selective OH− Adsorption for Durable Alkaline Seawater Oxidation at Industrial Current Densities
Source: Nanomicro Lett. 2026 Mar 18;18:288. doi: 10.1007/s40820-026-02133-8 (PMC12996582; doi:10.1007/s40820-026-02133-8)
Supplement: Supplementary file 1 — Supplementary file1 (DOCX 7073 KB) [file 40820_2026_2133_MOESM1_ESM.docx]

Supporting Information for

**Enhancing the Selective OH^–^ Adsorption for Durable Alkaline Seawater Oxidation at Industrial Current Densities**

Shangshu Hu^1^†, Jiao Yang^3^†, Yujuan Zhuang^1,2^, Xueyao Li^1,2^, Han Xu^4^, Fuwang Hu^1^, Zhishuo Yan^5^, Chao Liu^4^, Jianmin Yu^1^* and Lishan Peng^1,2^*

^1^ Key Laboratory of Rare Earths, Ganjiang Innovation Academy, Chinese Academy of Sciences, Ganzhou 341119, P. R. China

^2^ School of Rare Earths, University of Science and Technology of China, Hefei 230026, P. R. China

^3^ Institute of Applied Physics and Materials Engineering, University of Macau, Macao SAR, 999078, P. R. China

^4^ Jiangxi University of Science and Technology, Ganzhou 341000, P. R. China

^5^ Department of Electrical and Computer Engineering, North Dakota State University, Fargo 810052, USA

†Shangshu Hu and Jiao Yang contributed equally to this work.

*Corresponding authors. E-mail: [jmyu@gia.cas.cn](mailto:jmyu@gia.cas.cn) (Jianmin Yu); [lspeng@gia.cas.cn](mailto:lspeng@gia.cas.cn) (Lishan Peng)

**Supplementary Figures**

**
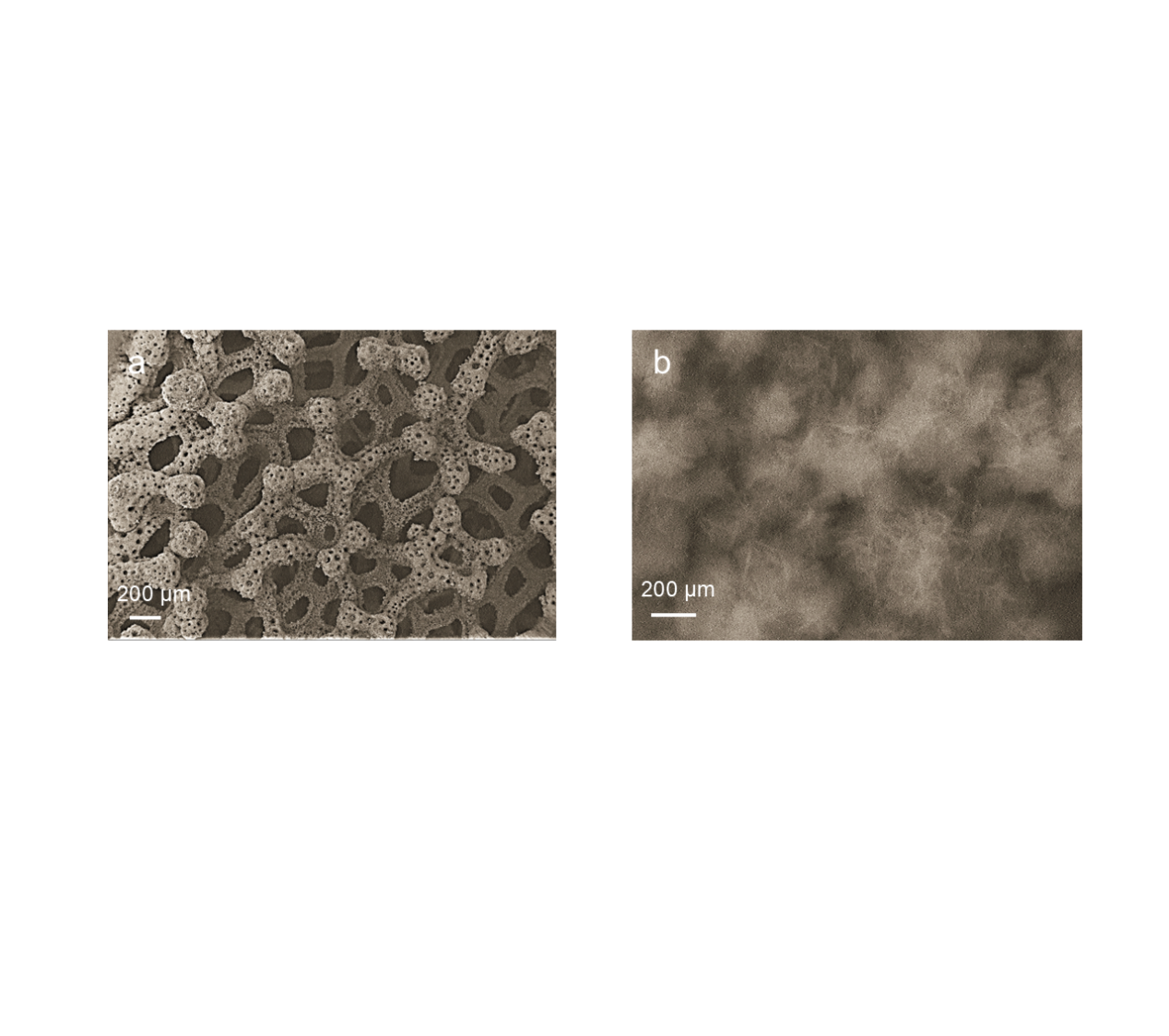
**

**Fig. S1** SEM images of NiFe-LDH


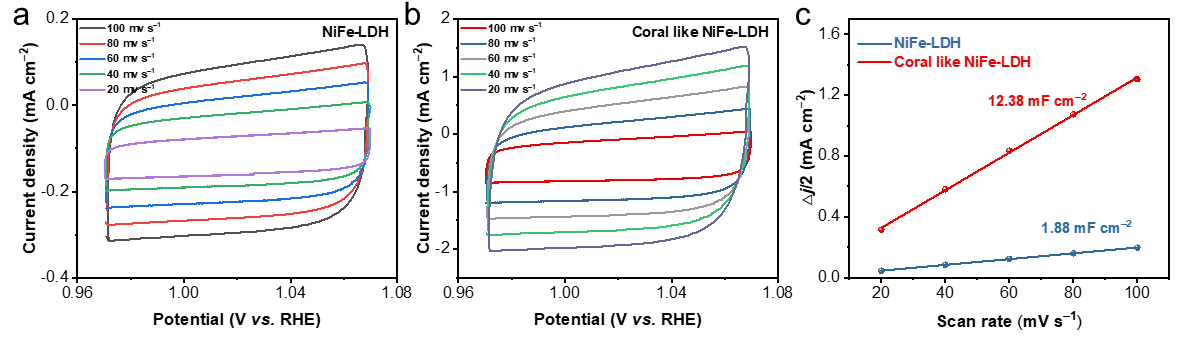


**Fig. S2** (**a, b**) CV curves of NiFe-LDH and coral-like NiFe-LDH, respectively. (**c**) Comparison of their *C*_dl_ derived from the CV measurements

**Note:** The Coral-like NiFe-LDH was fabricated via a hydrogen bubble-templated method [S1]. Its structural advantage is confirmed by the double-layer capacitance (*C*_dl_) measurement, which yields a value of 12.38 mF cm^–2^, markedly higher than the 1.88 mF cm^–2^ for the conventional NiFe-LDH. This drastically increased *C*_dl_ indicates a substantially larger electrochemical active surface area, which provides abundant nucleation sites for the NiFe-LDH growth, leading to a higher loading of the active phase.


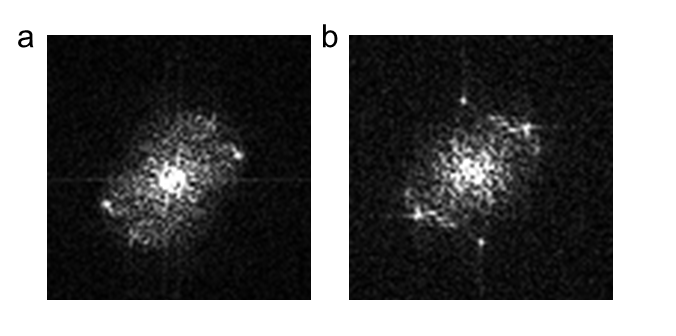


**Fig. S3** FFT images derived from the HRTEM images of (**a**) NiFe-LDH and (**b**) Ce(OH)CO_3_ area in NiFe-LDH/Ce(OH)CO_3_


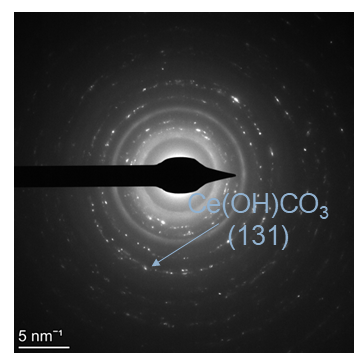


**Fig. S4** SAED pattern of NiFe-LDH/Ce(OH)CO_3_

**Note:** The discrete bright spots corresponding to the highly crystalline Ce(OH)CO_3_ phase, and broad diffraction rings originating from the poorly crystalline NiFe-LDH phase.


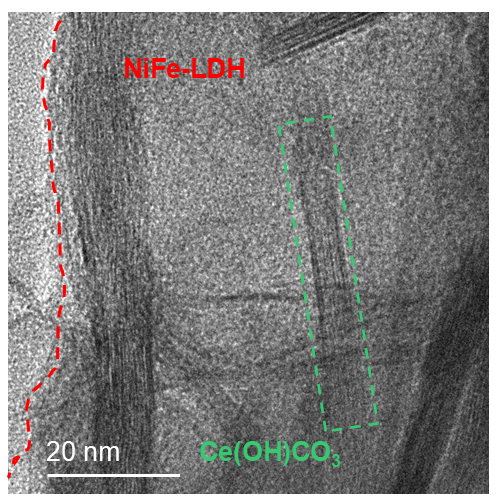


**Fig. S5** HRTEM images of NiFe-LDH/Ce(OH)CO_3_


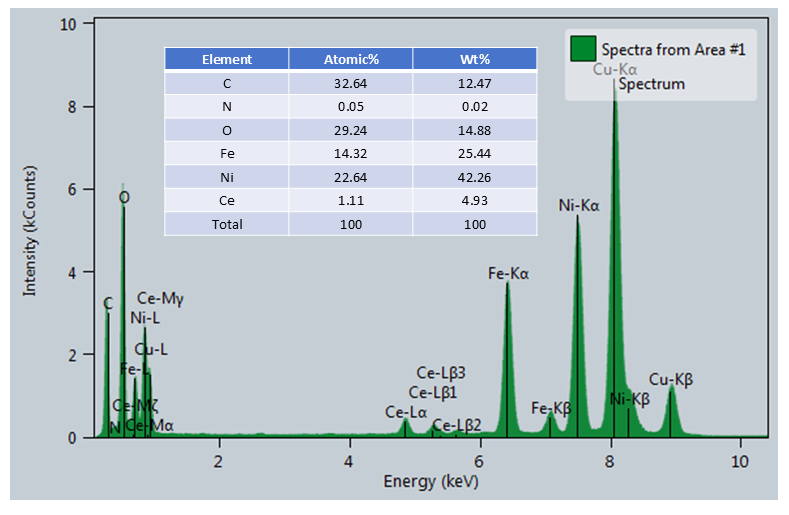


**Fig. S6** EDS spectrum and corresponding elemental composition (inset table) of the NiFe-LDH/Ce(OH)CO_3_ composite

**
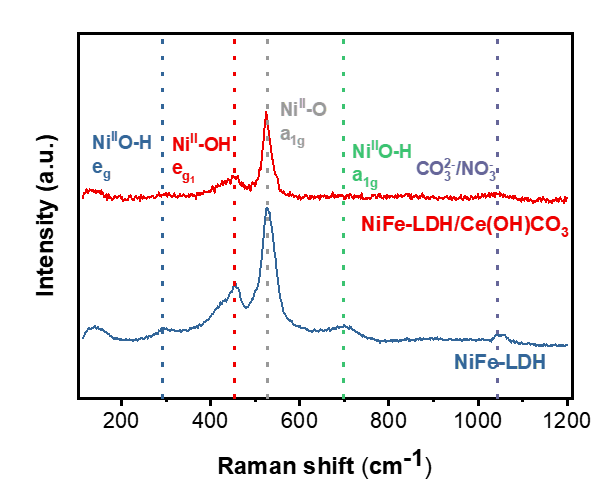
**

**Fig. S7** Raman spectra of NiFe-LDH and NiFe-LDH/Ce(OH)CO_3_

**Note:** CO_3_^2–^ intercalation in LDHs is known to enhance chloride resistance via two mechanisms: its high charge density strengthens layer bonding, and its small size reduces interlayer spacing, inhibiting Cl^–^ exchange [S2].

It is noteworthy that in our work, Raman analysis confirms the presence of intercalated CO_3_^2–^ in both the baseline and target catalysts. Since CO_3_^2–^ has the highest affinity for LDH interlayers (CO_3_^2–^ > SO_4_^2–^ > OH^–^ > Cl^–^ > NO_3_^–^) and becomes the dominant interlayer anion under our synthesis conditions [S3], it forms a consistent background in all samples. Thus, the superior performance of NiFe-LDH/Ce(OH)CO_3_ is not due to differential carbonate effects but to the specific role of Ce(OH)CO_3_ integration.


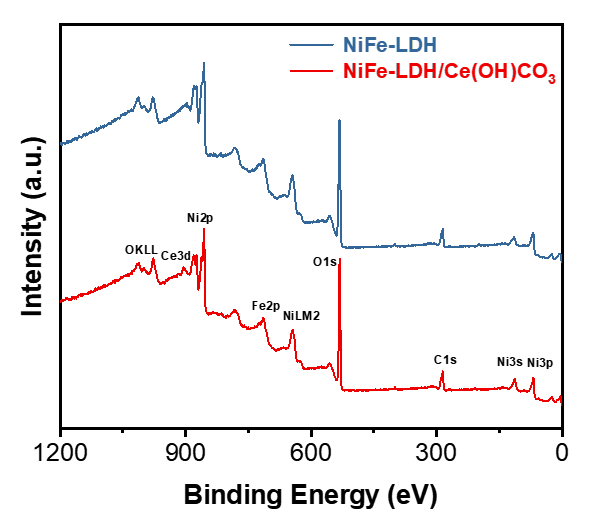


**Fig. S8** Survey XPS spectra of NiFe-LDH and NiFe-LDH/Ce(OH)CO_3_


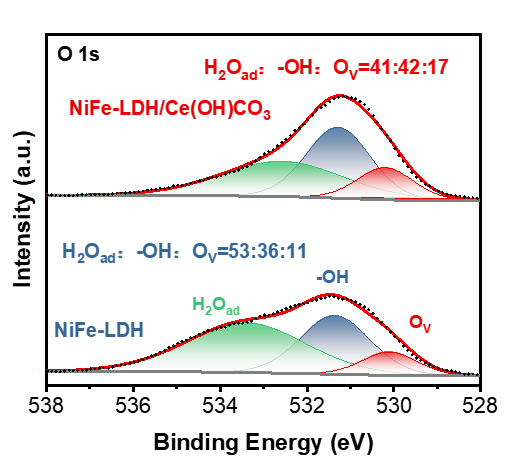


**Fig. S9** O 1s XPS spectra of NiFe-LDH and NiFe-LDH/Ce(OH)CO_3_


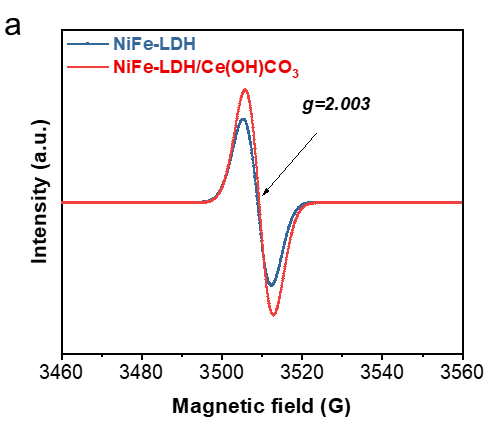


**Fig. S10** EPR spectra of NiFe-LDH and NiFe-LDH/Ce(OH)CO_3_ samples recorded at room temperature

**
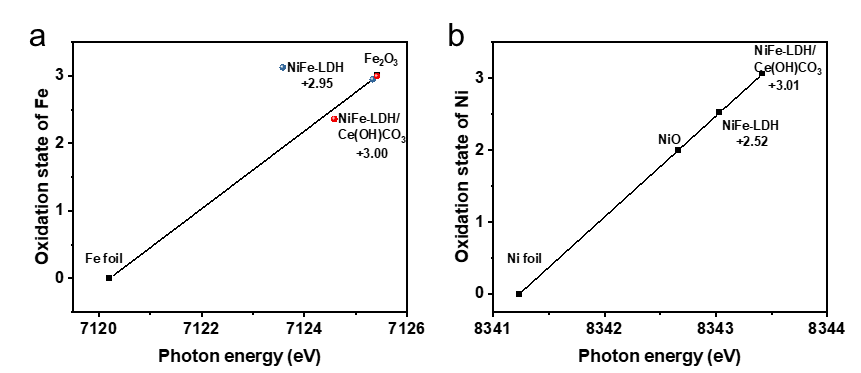
**

**Fig. S11** Oxidation States of (**a**) Fe and (**b**) Ni in NiFe-LDH and NiFe-LDH/Ce(OH)CO_3_ Determined by Linear Combination Fitting


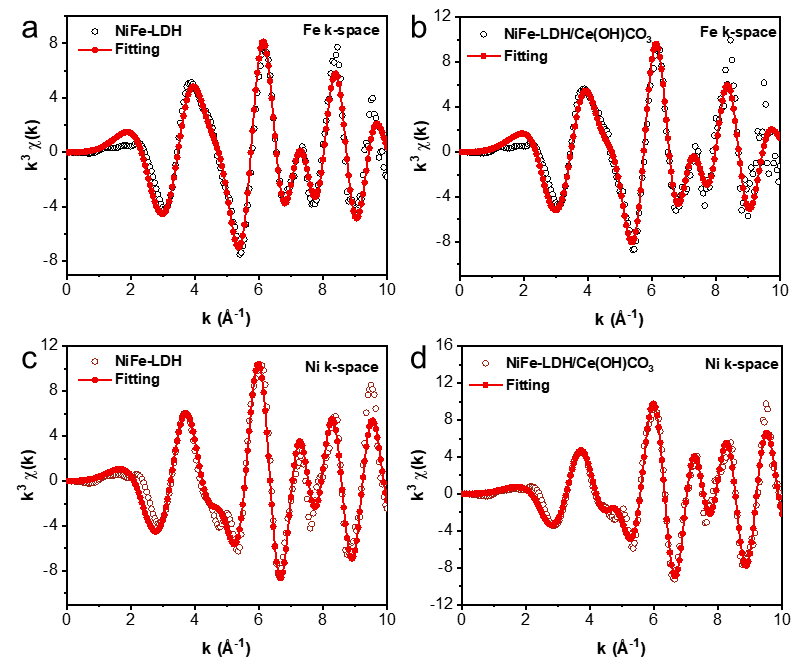


**Fig. S12** (**a, b**) Fe K-edge and (**c, d**) Ni K-edge XAFS spectra of NiFe-LDH and NiFe-LDH/Ce(OH)CO_3_


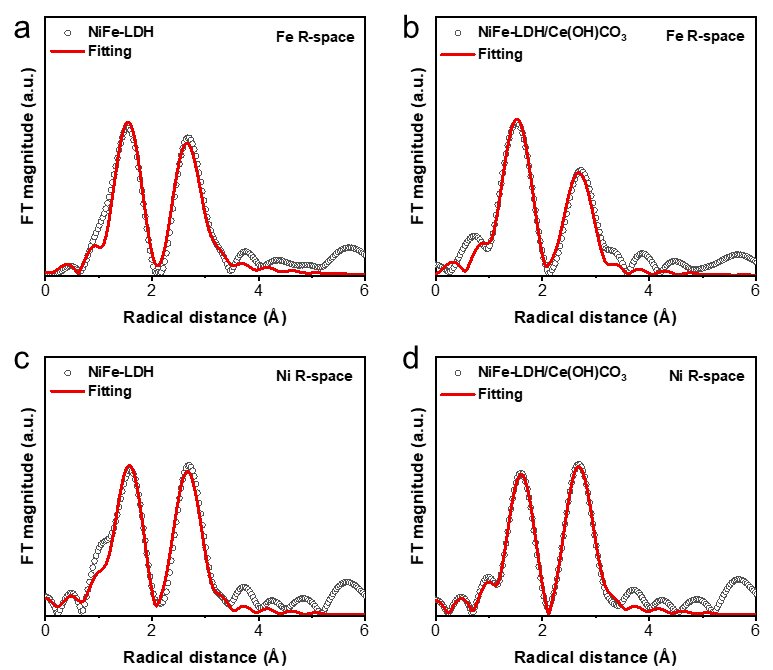


**Fig. S13** (**a, b**) Fe R-space and (**c, d**) R-space XAFS spectra of NiFe-LDH and NiFe-LDH/Ce(OH)CO_3_


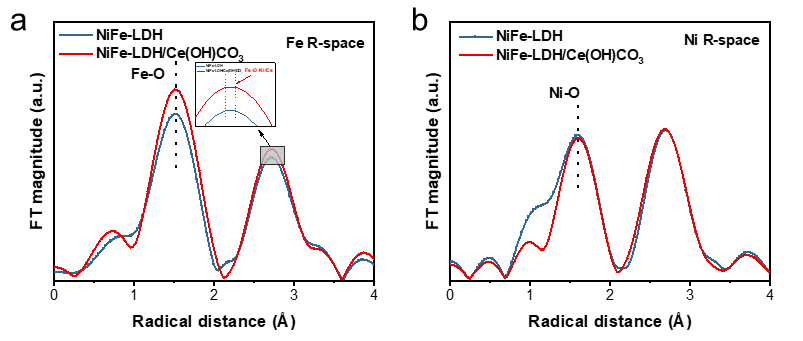


**Fig. S14** Fourier transforms of k^3^-weighted EXAFS spectra at (**a**) Fe and (**b**) Ni K-edges


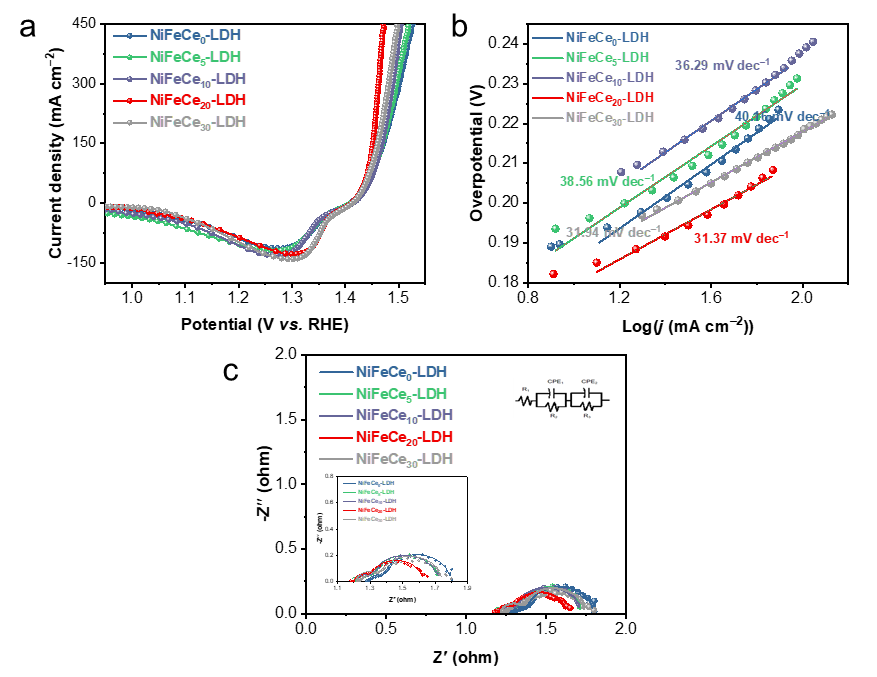


**Fig. S15** The OER activities of catalysts. (**a**) LSV polarization curves. (**b**) Tafel slopes derived from polarization curves. (**c**) EIS measurements

**Note：** The electrochemical activity of NiFeCe_0_-LDH, NiFeCe_5_-LDH, NiFeCe_10_-LDH, NiFeCe_20_-LDH, and NiFeCe_30_-LDH catalysts with different Ce doping amounts was compared. The results showed that NiFeCe_20_-LDH had the best electrochemical activity. This superior activity is ascribed to its significantly reduced interfacial resistance, as evidenced by its markedly lower charge-transfer resistance (*R*_ct_ = 0.322 Ω) compared to the other catalysts (*R*_ct_ ranging from 0.385 to 0.414 Ω). This minimal *R*_ct_ underpinned the highest charge-transfer efficiency and lowest overall impedance. Consequently, NiFeCe_20_-LDH was selected as the target catalyst (denoted as NiFe-LDH/Ce(OH)CO_3_) for subsequent investigations.


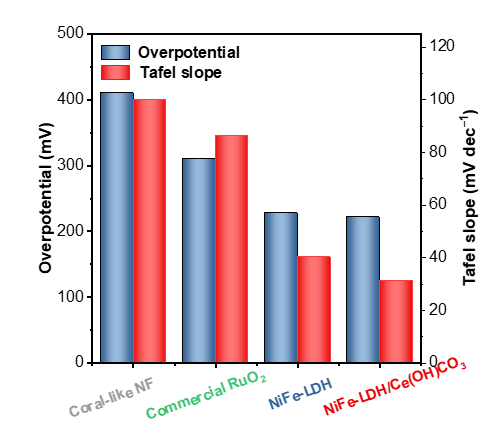


**Fig. S16** Overpotential of Coral-like NF, Commercial RuO_2_, NiFe-LDH/Ce(OH)CO_3,_ and NiFe-LDH


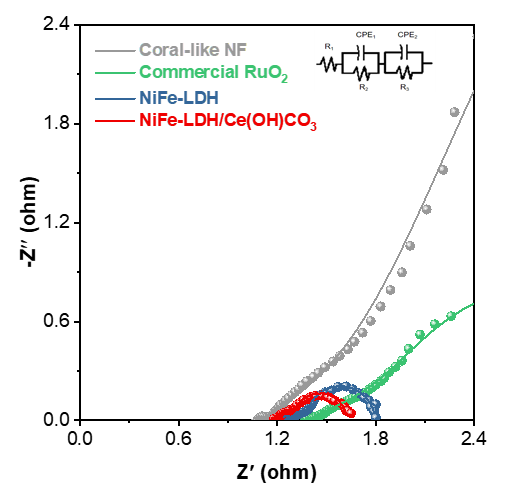


**Fig. S17** EIS measurements of Coral-like NF, Commercial RuO_2_, NiFe-LDH/Ce(OH)CO_3,_ and NiFe-LDH


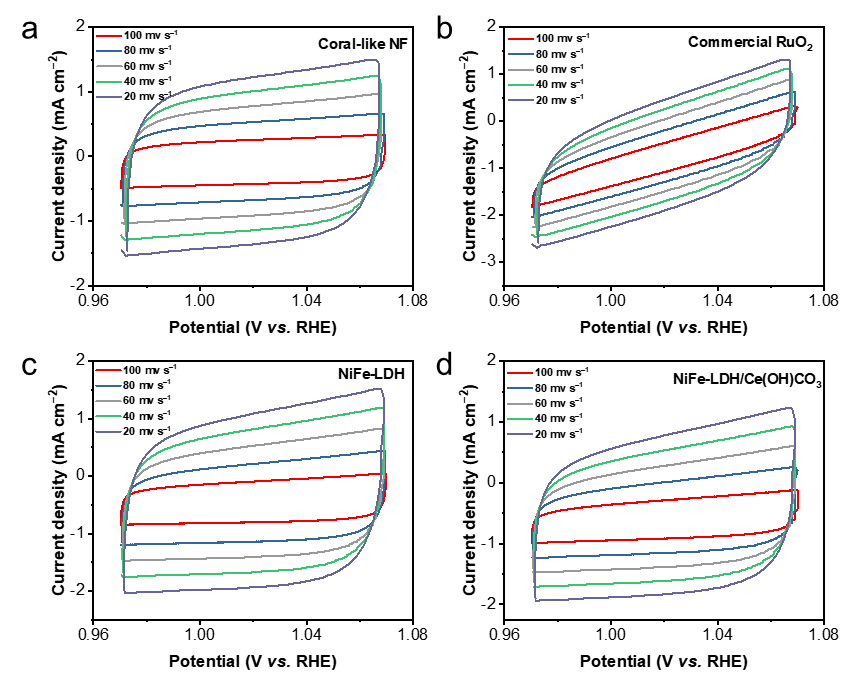


**Fig. S18** The CV curves of (**a**) coral-like NF, (**b**) commercial RuO_2_, (**c**) NiFe-LDH, and (**d**) NiFe-LDH/Ce(OH)CO_3_ at different scan rates

**
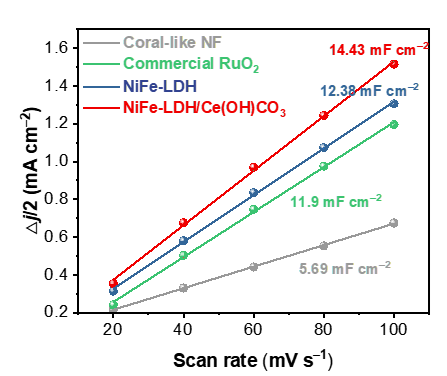
**

**Fig. S19** *C*_dl_ of coral-like NF, commercial RuO_2_, NiFe-LDH, and NiFe-LDH/Ce(OH)CO_3_


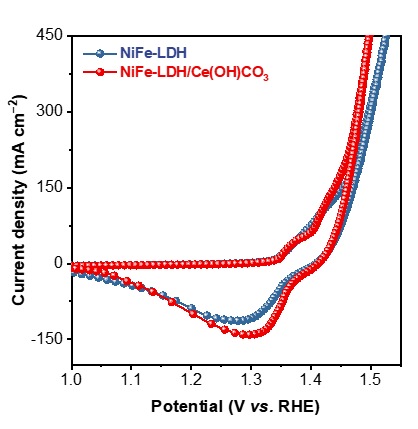


**Fig. S20** CV polarization curves for NiFe-LDH and NiFe-LDH/Ce(OH)CO_3_


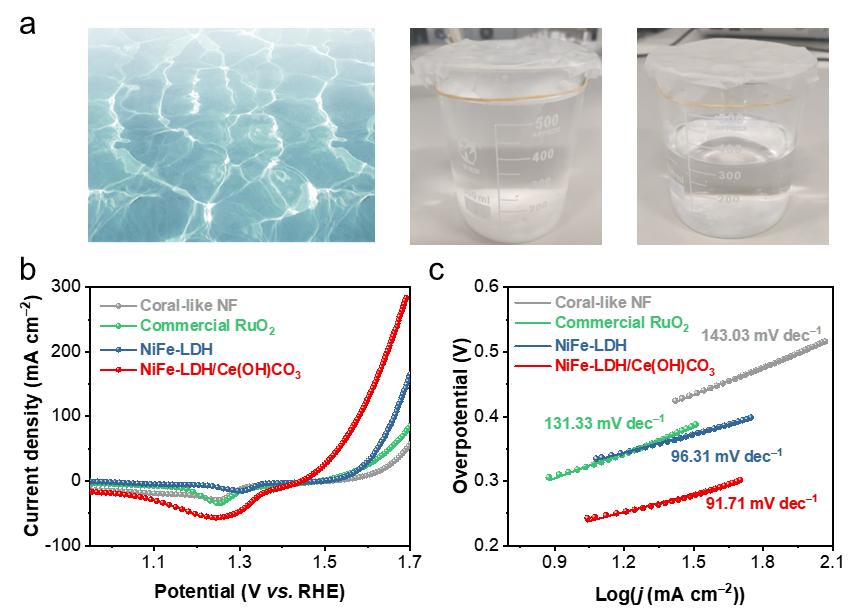


**Fig. S21** (**a**) Illustration of seawater pretreatment for alkaline seawater electrolysis. The OER activities of catalysts in alkaline natural seawater. (**b**) LSV polarization curves. (**c**) Tafel slopes derived from polarization curves

**Note:** All seawater used in this study was sourced from the Bohai Sea (36°58.0′N–40°59.0′N, 118°42.0′E–122°17.0′E, China). To utilize the seawater while removing interfering cations, 1 L of seawater was treated directly with 1 M KOH and allowed to stand for 12 hours to precipitate Ca^2+^/Mg^2+^ ions (as hydroxides). The resulting clear supernatant was then collected for electrolysis tests [S4].


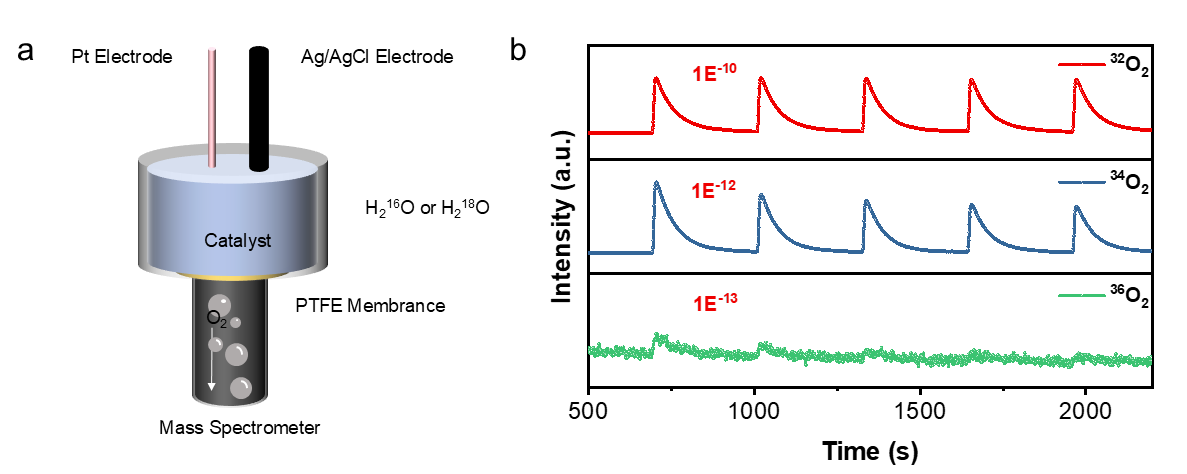


**Fig. S22** (a) Schematic of the DEMS electrochemical cell. (b). DEMS signals of NiFe-LDH/Ce(OH)CO_3_ for ^16^O^16^O, ^16^O^18^O, and ^18^O^18^O

**Note：** The catalyst was first electrochemically pretreated in H_2_^18^O electrolyte, followed by LSV. It was then transferred to an H_2_^16^O solution for further LSV measurements while DEMS continuously monitored the gaseous products. The DEMS results show that the mass spectrometric signal for oxygen increases with the applied potential, correlating with the rise in current density observed in the LSV, thus confirming that OER occurs on the catalyst surface [S5].


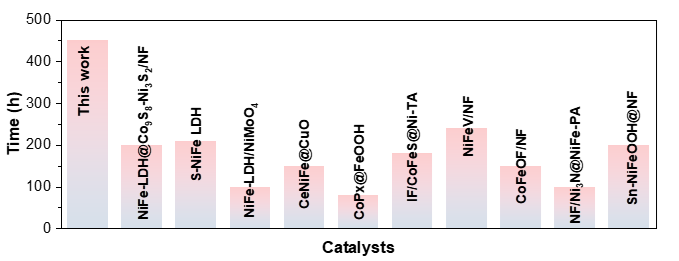
 **Fig. S23** Comparisons of state-of-the-art works of the stability with this work


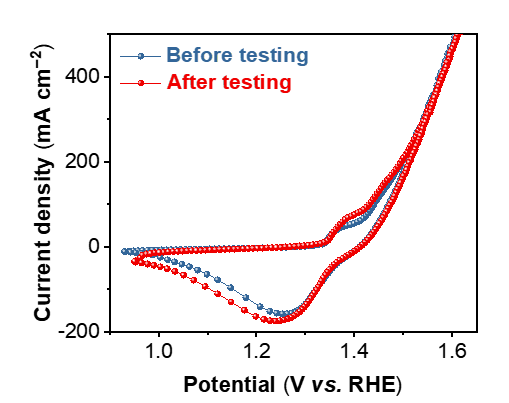


**Fig. S24** Comparison of CV Curves before and after Chronopotentiometry

**
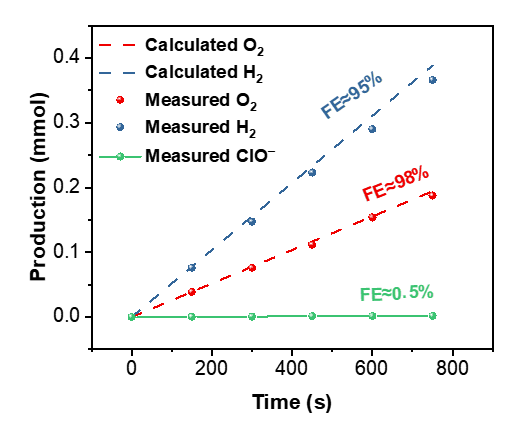
**

**Fig. S25** Faradaic efficiency tests of NiFe-LDH/Ce(OH)CO_3_ in alkaline seawater at the constant current densities of 0.1 A cm^−2^


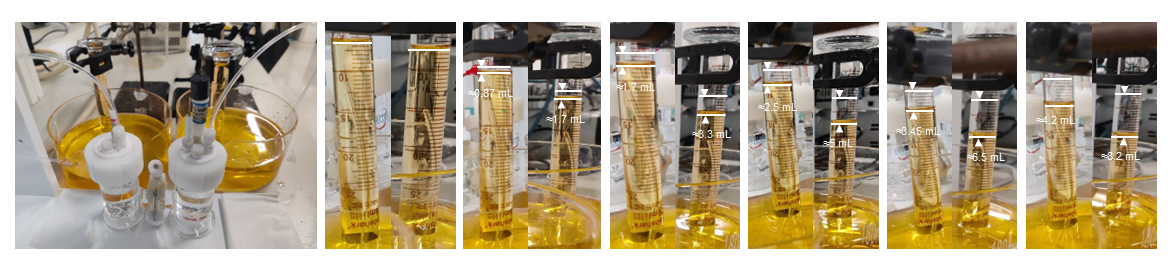


**Fig. S26** Corresponding digital photos of the typical drainage method


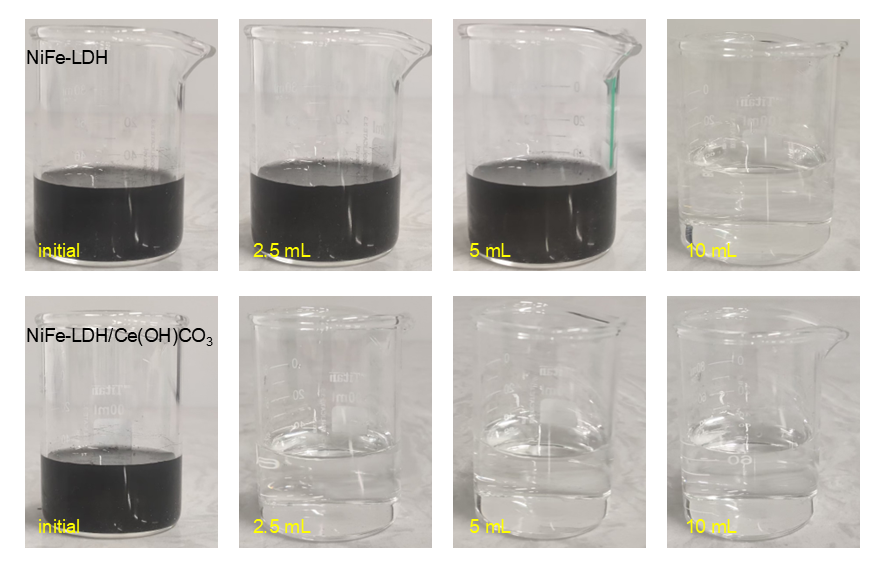


**Fig. S27** Corresponding photographs of iodometric titration after constant current test at a current density of 1 A cm^–2^

**Note：** Quantitative iodometric titration was performed on the electrolyte after stability testing under identical conditions. Upon acidification and addition of potassium iodide and starch indicator, the amount of Na_2_S_2_O_3_ (0.01 M) required to reach the titration endpoint was used to quantify the chlorine oxidation products. The NiFe‑LDH/Ce(OH)CO_3_ sample required only 2.5 mL to initiate fading of the blue color. In contrast, the NiFe‑LDH sample still showed blue color after 5 mL, but began fading only after 10 mL. This marked reduction in Na_2_S_2_O_3_ consumption for the composite directly confirms the effective suppression of chloride oxidation during prolonged operation.

**Supplementary methods**

**1. Water-Displacement Method and Faradaic Efficiency Calculation** [S6]

OER tests were conducted in a three-electrode system using a gas-tight electrochemical cell filled with alkaline simulated seawater. Galvanostatic measurements were performed at a current density of 100 mA cm⁻² to maintain a constant rate of oxygen production. During each 150‑second interval, the evolved oxygen and hydrogen gases were collected by the conventional water‑displacement method. It should be noted that any chlorine gas generated dissolves completely in the electrolyte and thus does not contribute to the collected gas volume. Methyl orange was added to the collection vessel to improve the visibility of the meniscus.

**2. Iodometric Titration** [S7]

For the determination of hypochlorite and chlorite (available chlorine) in the liquid phase, the following procedure was followed:

(i) 10 mL of electrolyte was collected after electrolysis at different current densities.

(ii) 10 mL of KI (10%) was added to the electrolyte.

(iii) 2 mL of 30% H_2_SO_4_ was added to the electrolyte.

(iv) The solution was titrated with 0.001 M Na_2_S_2_O_3_.

The reaction that occurs during determination is as follows:

ClO^–^ + 2I^–^ + 2H^+^ → Cl^–^ + I_2_ + H_2_O (S1)

2S_2_O_3_^2–^ + I_2_ → 2I^–^ + S_4_O_6_^2–^ (S2)

The selectivity of oxygen evolution was calculated by observing the volume of Na_2_S_2_O_3_ consumed at the end of the titration, which was repeated three times.


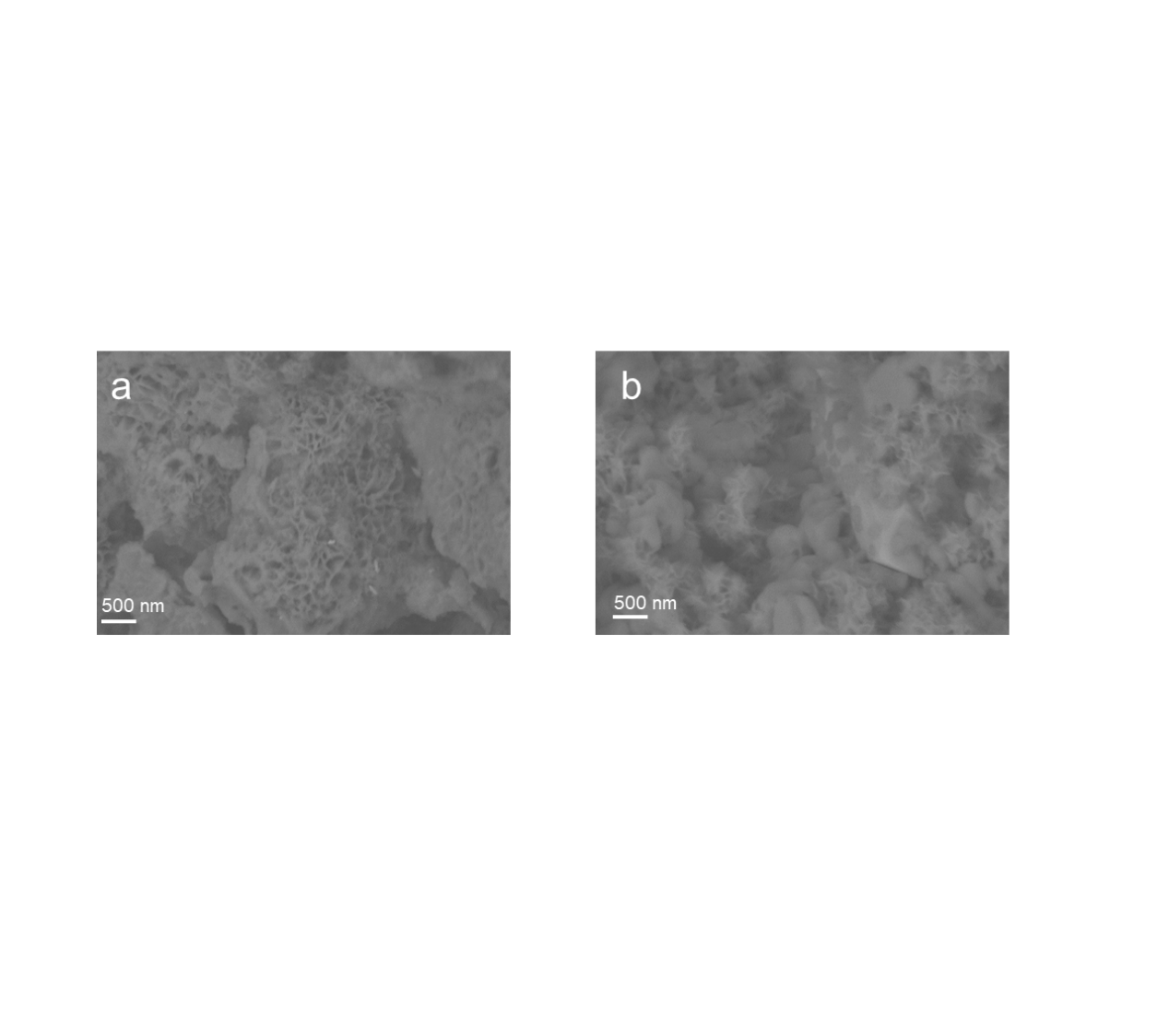


**Fig. S28** SEM images of NiFe-LDH (**a**) and NiFe-LDH/Ce(OH)CO_3_ (**b**) after constant current test


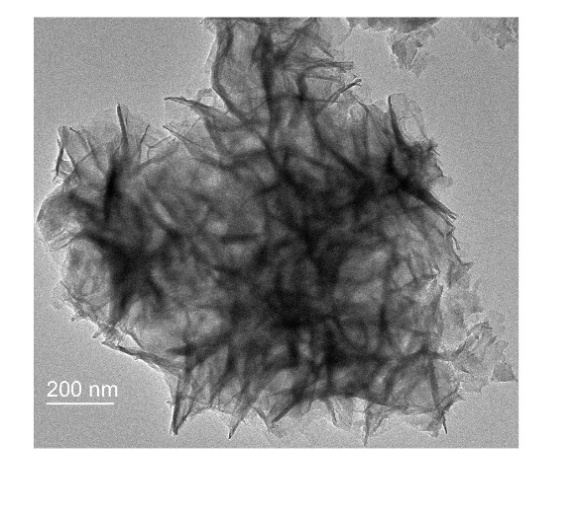


**Fig. S29** TEM image of NiFe-LDH/Ce(OH)CO_3_ after constant current test


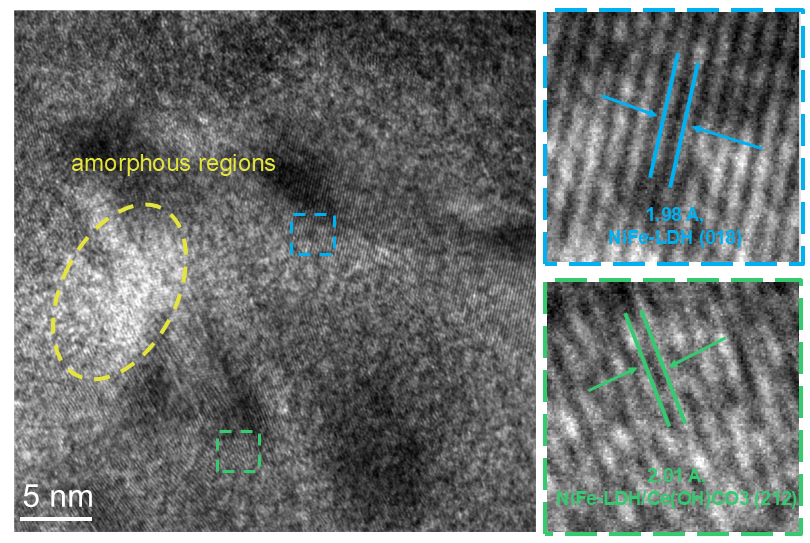


**Fig. S30** HRTEM images of NiFe-LDH/Ce(OH)CO_3_ after constant current test


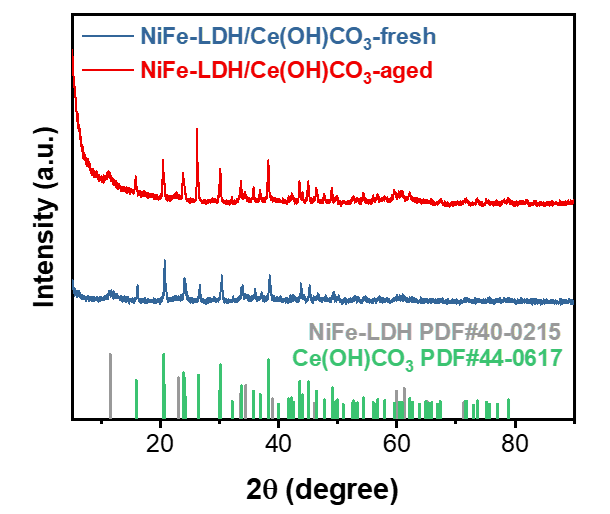


**Fig. S31** XRD patterns before and after the constant current test


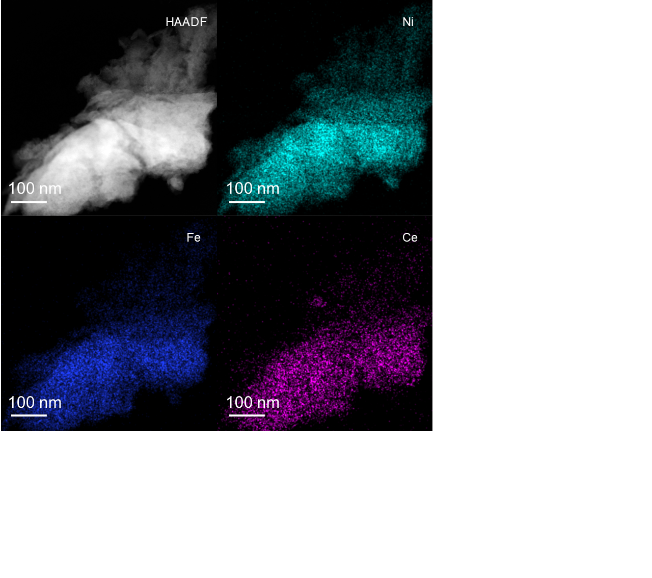


**Fig. S32** HAADF image and EDS images of NiFe-LDH/Ce(OH)CO_3_ after constant current test


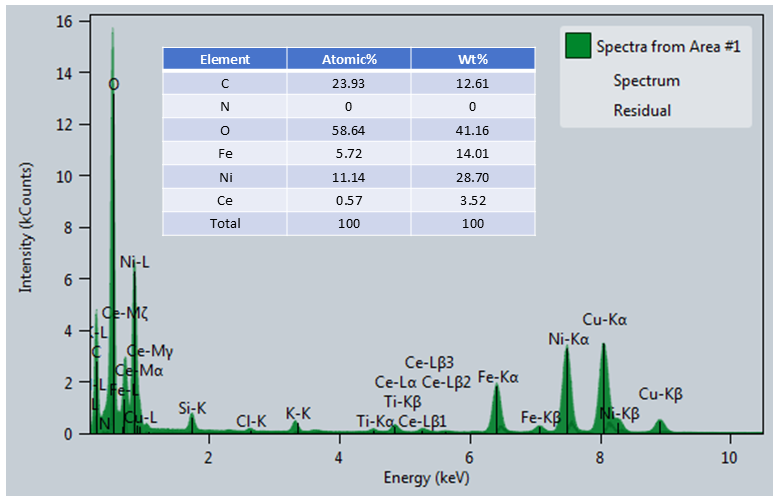


**Fig. S33** EDS spectrum and corresponding elemental composition (inset table) of the NiFe-LDH/Ce(OH)CO_3_ composite after constant current test


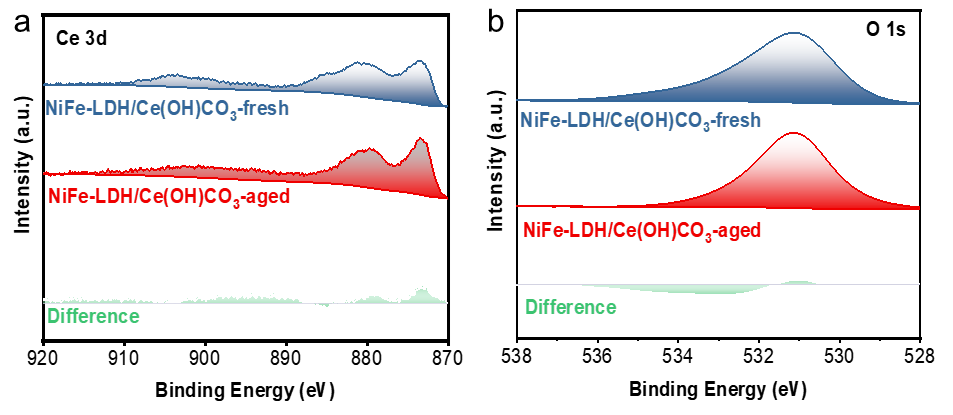


**Fig. S34** XPS spectra before and after the constant current test. (a) Ce 3d and (b) O 1s of NiFe-LDH/Ce(OH)CO_3_

**
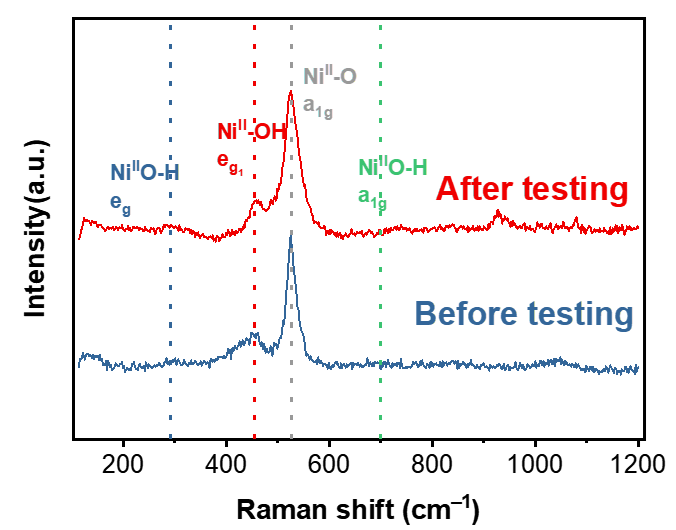
**

**Fig. S35** Raman spectra of NiFe-LDH/Ce(OH)CO_3_ before and after constant current test

**
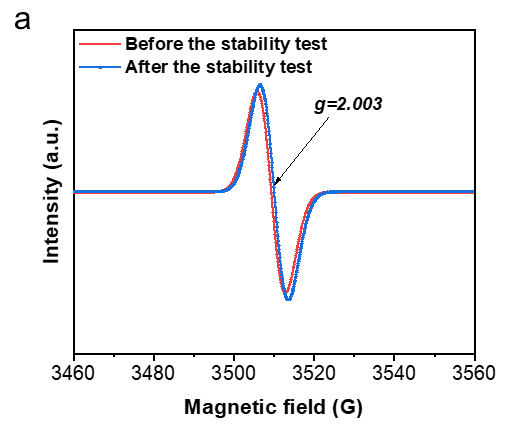
**

**Fig. S36** EPR spectra of NiFe-LDH/Ce(OH)CO_3_ before and after constant current test

**Note：** The lack of significant change in oxygen vacancy concentration before and after stability testing indicates that the O_v_ do not directly serve as active sites in the OER process.


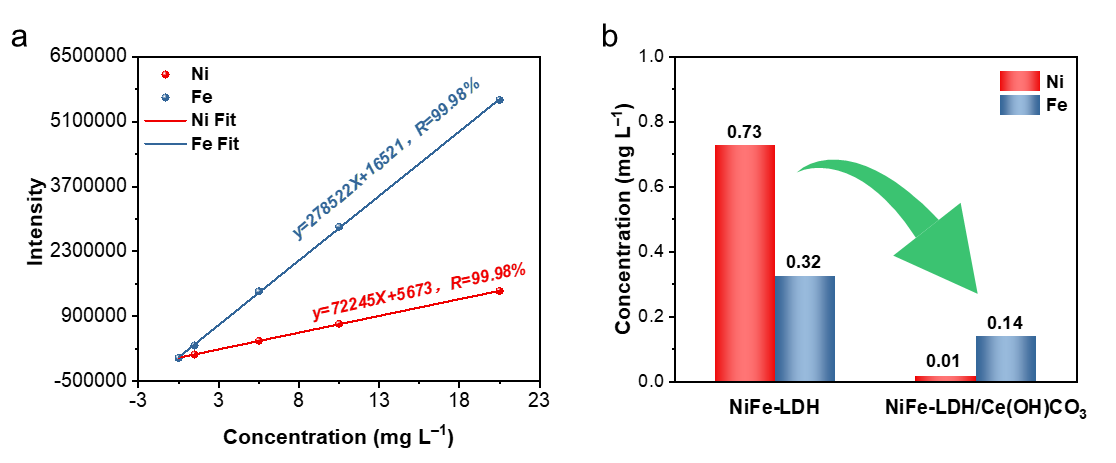


**Fig. S37** ICP analysis after constant current test. (**a**) Calibration curves for Ni and Fe. (**b**) Leaching amounts of Ni and Fe

**
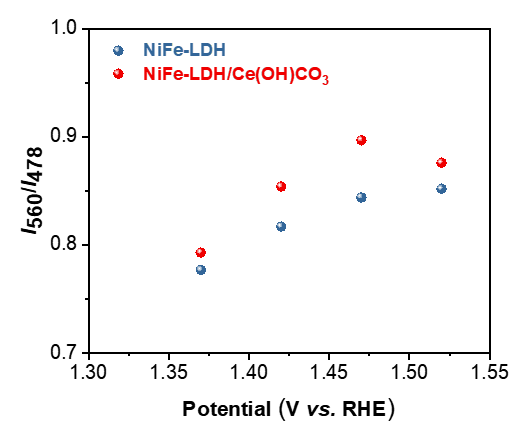
**

**Fig. S38** Comparison of ratios for band intensity (I_560_/I_478_)

**
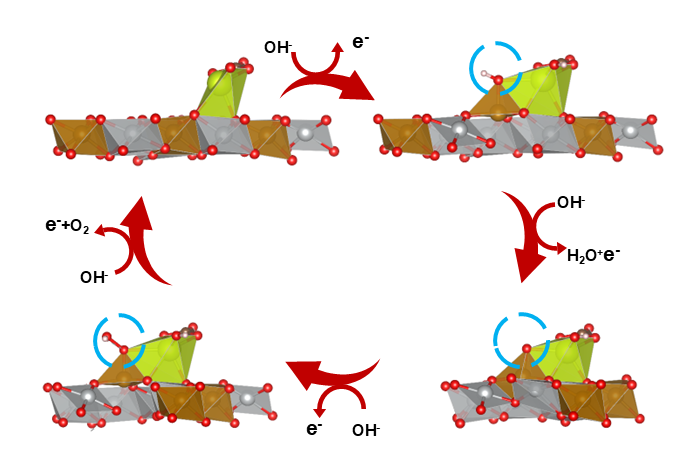
**

**Fig. S39** The OER pathway of NiFe-LDH/Ce(OH)CO_3_

**Note：**The OER on the NiFe-LDH/Ce(OH)CO_3_ surface proceeds via the adsorbate evolution mechanism (AEM). Under alkaline conditions, NiFe-LDH/Ce(OH)CO_3_ initially adsorbs OH⁻ to form an *OH intermediate, which undergoes dehydrogenation to yield *O. Subsequently, the *O species reacts with another OH⁻ through nucleophilic attack, generating an *OOH intermediate. Finally, O_2_ is released via *OOH desorption facilitated by additional OH⁻.


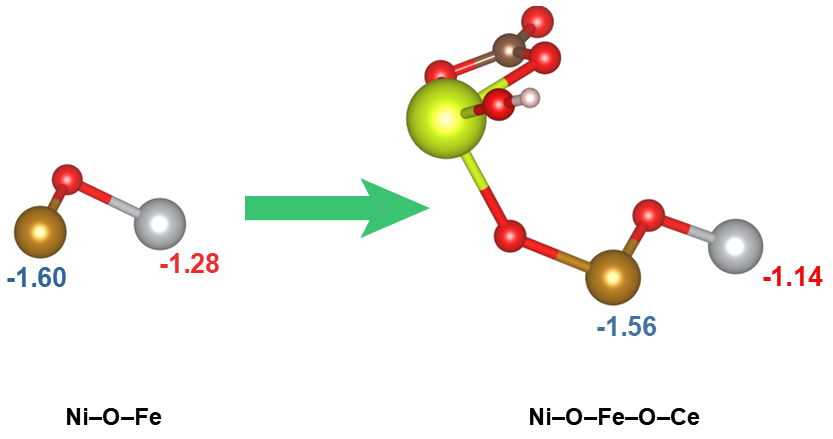


**Fig. S40** Schematic diagram of the Bader charge redistribution induced by incorporating Ce(OH)CO_3_ into NiFe-LDH

**
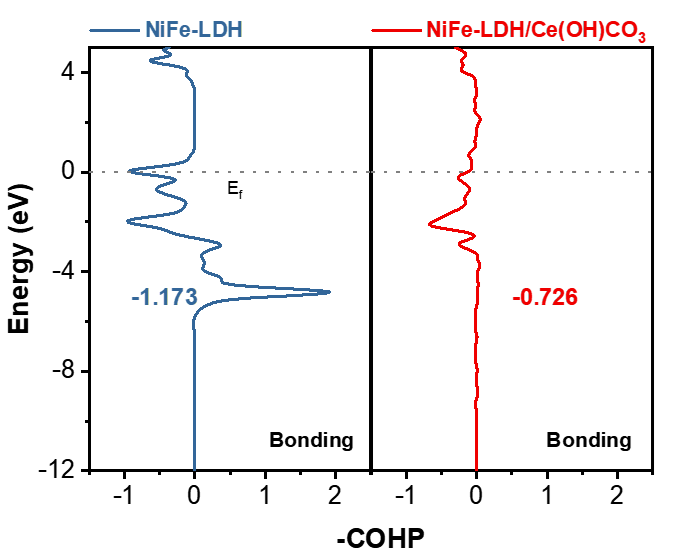
**

**Fig. S41** COHP analysis for NiFe-LDH and NiFe-LDH/Ce(OH)CO_3_ after *Cl absorption

**Note：**The integrated crystal orbital Hamiltonian population (ICOHP) analysis was employed to evaluate the binding affinity between the catalyst and Cl⁻. The ICOHP value for NiFe-LDH/Ce(OH)CO_3_ is –0.726 eV, in contrast to –1.173 eV for pristine NiFe-LDH. This notable reduction indicates fewer electrons available for bonding with Cl⁻ in the composite, which directly corroborates its enhanced resistance to chloride-induced corrosion.

**Table S1** Fe K-edge EXAFS fitting results

| **Sample** | **Path** | ***N*** | ***R* (Å)** | ***σ*^2^×10^3^ (Å^2^)** | **Δ*E* (eV)** | ***R* factor** |
| --- | --- | --- | --- | --- | --- | --- |
| NiFe-LDH | Fe-O | 5.80±1.12 | 1.99 | 8.09 | -2.12 | 0.017 |
|  | Fe-M | 5.75±2.54 | 3.07 | 10.78 |  |  |
| NiFe-LDH/Ce(OH)CO_3_ | Fe-O | 6.17±0.73 | 2.00 | 7.11 | -1.14 | 0.008 |
|  | Fe-M | 9.43±2.62 | 3.08 | 14.69 |  |  |

[a]: *k* range: 3-9.5 (Å^–1^); *R* range: 1-3.6 Å; *S*_0_^2^ = 0.95

*N*: coordination numbers; *R*: bond distance; *σ*^2^: Debye-Waller factors; Δ*E*: the inner potential correction. *R* factor: goodness of fit. *Fitting with fixed parameter. The number of variable parameters is 7.

**Note:** Fe K-edge and Ni K-edge XAFS and EXAFS data were collected in fluorescence mode on the X-ray Absorption Spectroscopy beamline at the Ganjiang Innovation Academy (Chinese Academy of Sciences). The powder samples were homogeneously mixed with cellulose to obtain a metal concentration of around 10000 ppm. A Si(111) single crystal was used to monochromatize the X-ray beam. The raw XAFS data were processed (background-subtraction, normalization, and Fourier transformation) using standard procedures within the Athena module of the IFEFFIT software package. EXAFS fitting was performed by the Artemis module, following the EXAFS equation below:


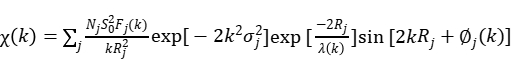
 (S3)

where *S_0_^2^* is the amplitude reduction factor, *Fj(k)* is the effective curved-wave backscattering amplitude, *N_j_* is the number of neighbors in the *j_th_* atomic shell, *R_j_* is the distance between the X-ray absorbing central atom and the atoms in the *j_th_* atomic shell, *λ* is the mean free path in Å, *ϕ_j_(k)* is the phase shift, *σ_j_*^2^ is the Debye-Waller parameter of the *j_th_* atomic shell (variation of distances around the average *R_j_*) [S8].

**Table S2** Ni K-edge EXAFS fitting results

| **Sample** | **Path** | ***N*** | ***R* (Å)** | ***σ*^2^×10^3^ (Å^2^)** | **Δ*E* (eV)** | ***R* factor** |
| --- | --- | --- | --- | --- | --- | --- |
| NiFe-LDH | Ni-O | 6.34±1.16 | 2.05 | 6.34 | -2.01 | 0.016 |
|  | Ni-M | 6.16±2.18 | 3.09 | 6.16 |  |  |
| NiFe-LDH/Ce(OH)CO_3_ | Ni-O | 6.84±0.64 | 2.06 | 6.84 | -0.48 | 0.004 |
|  | Ni-M | 5.68±0.91 | 3.10 | 5.68 |  |  |

[a]: *k* range: 3-9.5 (Å^–1^); *R* range: 1-3.6 Å; *S*_0_^2^ = 0.95

*N*: coordination numbers; *R*: bond distance; *σ*^2^: Debye-Waller factors; Δ*E*: the inner potential correction. *R* factor: goodness of fit. *Fitting with fixed parameter. The number of variable parameters is 7.

**Table S3** EIS fitting data for NiFeCe_0_-LDH, NiFeCe_5_-LDH, NiFeCe_10_-LDH, NiFeCe_20_-LDH, and NiFe_30_-LDH

| Samples | *R*_s_ (Ω) | *R*_rt_ (Ω) | *R*_ct_ (Ω) |
| --- | --- | --- | --- |
| NiFeCe_0_-LDH | 1.280 | 0.098 | 0.414 |
| NiFeCe_5_-LDH | 1.208 | 0.107 | 0.403 |
| NiFeCe_10_-LDH | 1.207 | 0.101 | 0.400 |
| NiFeCe_20_-LDH | 1.201 | 0.100 | 0.322 |
| NiFeCe_30_-LDH | 1.218 | 0.138 | 0.385 |

**Note：**The incorporation of Ce(OH)CO_3_ likely increases the concentration of oxygen vacancies, which consequently enhances both the OER activity and electrical conductivity (reflected in a lower *R*_s_) of NiFe-LDH across different Ce(OH)CO_3_ loading levels.

**Table S4** EIS fitting data for coral-like NF, commercial RuO_2_, NiFe-LDH, and NiFe-LDH/Ce(OH)CO_3_

| Samples | *R*_s_ (Ω) | *R*_rt_ (Ω) | *R*_ct_ (Ω) |
| --- | --- | --- | --- |
| Coral-like NF | 1.173 | 0.736 | 13.950 |
| Commercial RuO_2_ | 1.431 | 0.298 | 1.550 |
| NiFe-LDH | 1.280 | 0.098 | 0.414 |
| NiFe-LDH/Ce(OH)CO_3_ | 1.201 | 0.100 | 0.322 |

**Table S5** Comparison of OER performance between NiFe-LDH/Ce(OH)CO_3_ and reported catalysts

| Catalysts | Overpotential (mV) | Tafel slope (mV dec^–1^) | Refs. |
| --- | --- | --- | --- |
| NiFe-LDH/Ce(OH)CO_3_ | 221 | 31.37 | This work |
| FeMoO_4_/NF | 271 | 41.4 | [S9] |
| AlCoCrFeNi-HEAS | 332 | 48.87 | [S10] |
| NiFe-LDH-S350 | 302 | 50.82 | [S11] |
| NNSN/NF | 296 | 215 | [S12] |
| NiO/Ni_3_S_2_@Ni_5_P_4_ | 340 | 69.9 | [S13] |
| ZnFe-BDC-0.75 | 308 | 47.72 | [S14] |
| FeOOH/CoFe-LDH | 240 | 55.6 | [S15] |
| FeOOH-NiCoMoLDH/NF | 256 | 59.39 | [S16] |
| S-NiFeO_x_H_y_/CC | 331 | 63 | [S17] |

**Note:** The electrolysis was performed in an aqueous electrolyte of 1 M KOH + 0.5 M NaCl.

**Table S6** Comparison of stability performance between NiFe-LDH/Ce(OH)CO_3_ and reported catalysts

| Catalysts | Time (h) | Current density (mA cm^–1^) | Electrolyte | Refs. |
| --- | --- | --- | --- | --- |
| NiFe-LDH/Ce(OH)CO_3_ | 450 | 1000 | Simulated alkaline seawater | This work |
| NiFe-LDH@Co_9_S_8_-Ni_3_S_2_/NF | 200 | 500 | Simulated alkaline seawater | [S18] |
| S-NiFe | 210 | 500 | Alkaline seawater | [S19] |
| NiFe-LDH/NiMoO_4_ | 100 | 1000 | Alkaline seawater | [S20] |
| CeNiFe@CuO | 150 | 500 | Alkaline seawater | [S21] |
| CoPx@FeOOH | 80 | 500 | Alkaline seawater | [S22] |
| IF/CoFeS@Ni-TA | 180 | 500 | Alkaline seawater | [S23] |
| NiFeV/NF | 240 | 500 | Alkaline seawater | [S24] |
| CoFeOF/NF | 150 | 400 | Simulated alkaline seawater | [S25] |
| NF/Ni_3_N@NiFe-PA | 100 | 500 | Alkaline seawater | [S26] |
| Sn-NiFeOOH@NF | 200 | 500 | Simulated alkaline seawater | [S27] |

**Table S7** Calculation of efficiency and H_2_ cost for the NiFe-LDH/Ce(OH)CO_3_-based AEM electrolyzer at various current densities in 1.0 M KOH + 0.5 M NaCl

| j (A cm^–2^) | Voltage (V) | H_2_ production rate (mol H_2_ cm^–2^ s^–1^) | H_2_ power out (W cm^–2^) | Electrolyzer Power (W cm^–2^) | Efficiency of AEM (%) | Price per GGE H_2_ ($) |
| --- | --- | --- | --- | --- | --- | --- |
| 0.1 | 1.62 | 5.18 × 10^-7^ | 0.125 | 0.162 | 77.16 | 0.86 |
| 0.2 | 1.71 | 1.04 × 10^-6^ | 0.251 | 0.342 | 73.39 | 0.91 |
| 0.3 | 1.76 | 1.56 × 10^-6^ | 0.378 | 0.528 | 71.59 | 0.94 |
| 0.4 | 1.80 | 2.07 × 10^-6^ | 0.501 | 0.721 | 69.44 | 0.96 |
| 0.5 | 1.83 | 2.59 × 10^-6^ | 0.627 | 0.914 | 68.59 | 0.97 |
| 0.6 | 1.85 | 3.11 × 10^-6^ | 0.752 | 1.110 | 67.57 | 0.99 |
| 0.7 | 1.87 | 3.63 × 10^-6^ | 0.877 | 1.306 | 67.15 | 1.00 |
| 0.8 | 1.88 | 4.14 × 10^-6^ | 1.003 | 1.503 | 66.73 | 1.00 |
| 0.9 | 1.90 | 4.66 × 10^-6^ | 1.128 | 1.706 | 66.12 | 1.01 |
| 1.0 | 1.92 | 5.18 × 10^-6^ | 1.250 | 1.917 | 65.21 | 1.03 |

**Supplementary testing conditions**

For the anode, the self-supporting electrode was used directly, which consisted of a ≈1 μm-thick NF loaded with NiFe-LDH/Ce(OH)CO_3_ via a hydrothermal method. For the cathode, a commercial Raney Ni electrode was employed. The MEA was fabricated using an AEM (PAP-TP-85, 40 μm) pretreated by soaking in 1 M KOH for 24 h. The catalyst-coated electrodes were placed on both sides of the membrane to ensure intimate contact. Electrolysis tests were performed in a custom two-compartment cell with an active geometric area of 1 cm^2^. The anode was supplied with alkaline simulated seawater (1 M KOH + 0.5 M NaCl). Both electrolytes were deaerated and circulated at a flow rate of 10 mL min^–1^. All experiments were conducted at 80 °C. Polarization curves were recorded at a scan rate of 5 mV s^–1^, and stability tests were carried out under galvanostatic conditions (500 mA cm^–2^) with periodic iR compensation.

**Note:** These calculations consider only the electricity costs, following the method outlined in the literature. We explicitly use the lower heating value (LHV) of hydrogen:

H_2_ production rate@0.1 A cm^–2^

= (j A cm^–2^)(1 e^–^/1.602 × 10^–19^ C)(1 H_2_/2 e^–^)

= 0.1 A cm^–2^ / (1.602 × 10^–19^ C × 2) = 3.12 × 10^17^ mol H^2^ cm^-2^ s^-1^

LHV of H_2_

= 120 kJ g^–1^ H_2_ = 2.42 × 10^5^ J mol^–1^ H_2_

H_2_ power out

= (5.18× 10^–7^ mol cm^–2^ s^–1^) × (2.42 × 10^5^ J mol^–1^) = 0.125 W cm^–2^

Electrolyzer Power @ 0.1 A cm^–2^

= (0.1 A cm^–2^) (1.62V) = 0.162 W cm^–2^

Efficiency of AEM

= (H_2_ Power Out) / (Electrolyzer Power) = 0.125 W cm^–2^/0.162W cm^–2^ = 77.16%

Price per gasoline-gallon equivalent (GGE) H_2_

= 1 GGE H_2_/H_2_ production rate × Electrolyzer power × Electricity bill

= 0.997 kg / (5.18× 10^–7^ mol H_2_.cm^–2^ s^–1^ × 2 kg/mol) × 0.162 W cm^–2^ × $ 0.02 / kW h= $ 0.86 /GGE H_2_

**Table S8** Calculation of efficiency and H_2_ cost for the RuO_2_-based AEM electrolyzer at various current densities in 1.0 M KOH + 0.5 M NaCl

| j (A cm^–2^) | Voltage (V) | H_2_ production rate (mol H_2_ cm^–2^ s^–1^) | H_2_ power out (W cm^–2^) | Electrolyzer Power (W cm^–2^) | Efficiency of AEM (%) | Price per GGE H_2_ ($) |
| --- | --- | --- | --- | --- | --- | --- |
| 0.1 | 1.79 | 5.18 × 10^-7^ | 0.125 | 0.179 | 69.83 | 0.96 |
| 0.2 | 1.93 | 1.04 × 10^-6^ | 0.251 | 0.386 | 65.03 | 1.00 |
| 0.3 | 2.00 | 1.56 × 10^-6^ | 0.378 | 0.602 | 62.79 | 1.07 |
| 0.4 | 2.05 | 2.07 × 10^-6^ | 0.501 | 0.821 | 61.02 | 1.10 |
| 0.5 | 2.10 | 2.59 × 10^-6^ | 0.627 | 1.052 | 59.60 | 1.12 |
| 0.6 | 2.14 | 3.11 × 10^-6^ | 0.752 | 1.286 | 58.48 | 1.14 |
| 0.7 | 2.19 | 3.63 × 10^-6^ | 0.877 | 1.530 | 57.32 | 1.17 |

**Supplementary References**

1. R. Xiang, C. Tong, Y. Wang, L. Peng, Y. Nie et al., Hierarchical coral-like FeNi(OH) x/Ni *via* mild corrosion of nickel as an integrated electrode for efficient overall water splitting. Chin. J. Catal. **39**(11), 1736–1745 (2018). <https://doi.org/10.1016/S1872-2067(18)63150-X>
2. R. Fan, C. Liu, Z. Li, H. Huang, J. Feng et al., Ultrastable electrocatalytic seawater splitting at ampere-level current density. Nat. Sustain. **7**(2), 158–167 (2024). <https://doi.org/10.1038/s41893-023-01263-w>
3. N. Iyi, T. Matsumoto, Y. Kaneko, K. Kitamura, Deintercalation of carbonate ions from a hydrotalcite-like compound:   enhanced decarbonation using acid–salt mixed solution. Chem. Mater. **16**(15), 2926–2932 (2004). <https://doi.org/10.1021/cm049579g>
4. F.O. Boakye, F.U. Zaman, H. Zhang, A. Saeed, F.T. Dajan et al., Functional interface optimization strategy for Fe_3_Se_4_/NiSe_2_ anchored on MXene for ultrastable seawater splitting at industrial-level current density. Adv. Funct. Mater. **35**(32), 2424718 (2025). <https://doi.org/10.1002/adfm.202424718>
5. X. Tao, L. Hou, X. Wang, J. Jin, H. Li et al., Iron and oxygen vacancies co-modulated adsorption evolution and lattice oxygen dual-path mechanism for water oxidation. Nat. Commun. **16**(1), 8788 (2025). <https://doi.org/10.1038/s41467-025-63844-x>
6. N.-N. Liang, D.J. Kim, Z. Qiu, Y. Kweon, T.W. Kim et al., Defective antifluorite MnO_2_-layered RuO_2_ for direct seawater electrolysis at circum-neutral pH. Small **21**(35), 2504249 (2025). <https://doi.org/10.1002/smll.202504249>
7. R. Yuan, C. Liao, L. Cao, D. Li, S. Sun et al., Highly efficiency seawater electrolysis guided by coordinating catalysis of oxygen evolution reaction. Adv. Funct. Mater. **36**(3), e08413 (2026). <https://doi.org/10.1002/adfm.202508413>
8. L. Peng, N. Yang, Y. Yang, Q. Wang, X. Xie et al., Atomic cation-vacancy engineering of NiFe-layered double hydroxides for improved activity and stability towards the oxygen evolution reaction. Angew. Chem. Int. Ed. **60**(46), 24612–24619 (2021). <https://doi.org/10.1002/anie.202109938>
9. J. Tang, S. Sun, X. He, H. Zhang, C. X. Yang et al., An amorphous FeMoO_4_ nanorod array enabled high-efficiency oxygen evolution electrocatalysis in alkaline seawater. Nano Res*.* **17**, 2270-2275 (2023). https://doi.org 10.1007/s12274-023-6087-y
10. Z. Chen, K. Huang, B. Zhang, J. Xia, J. Wu et al., Corrosion engineering on AlCoCrFeNi high-entropy alloys toward highly efficient electrocatalysts for the oxygen evolution of alkaline seawater. Int. J. Miner. Metall. Mater. **30**(10), 1922–1932 (2023). <https://doi.org/10.1007/s12613-023-2624-7>
11. S.Y. Jung, S. Kang, K.M. Kim, S. Mhin, J.C. Kim et al., Sulfur-incorporated nickel-iron layered double hydroxides for effective oxygen evolution reaction in seawater. Appl. Surf. Sci. **568**, 150965 (2021). <https://doi.org/10.1016/j.apsusc.2021.150965>
12. J. Liang, Z. Zhao, Z. Su, W. Qu, R. Guo et al., Multiphase interface coupling of Ni-based sulfide composites for high-current-density oxygen evolution electrocatalysis in alkaline freshwater/simulated seawater/seawater. Dalton Trans. **53**(36), 15040–15047 (2024). <https://doi.org/10.1039/d4dt01673d>
13. Y. Yu, X. Chen, J. Li, Y. Xiao, X. Shi et al., Ni-based heterostructure with protective phosphide layer to enhance the oxygen evolution reaction for the seawater electrolysis. Int. J. Hydrog. Energy **51**, 1373–1380 (2024). <https://doi.org/10.1016/j.ijhydene.2023.07.282>
14. Y. Cheng, Y. Luo, Y. Zheng, J. Pang, K. Sun et al., Self-supporting one-dimensional ZnFe-BDC for electrocatalysis oxygen evolution reaction in alkaline and natural seawater. Int. J. Hydrog. Energy **47**(84), 35655–35665 (2022). <https://doi.org/10.1016/j.ijhydene.2022.08.138>
15. D. Wang, L. Liu, Y. Liu, W. Luo, Y. Xie et al., Hierarchical superaerophobic nanoarray electrode (FeOOH/CoFe LDH) as an efficient oxygen evolution reaction catalyst for alkaline seawater electrolysis. ACS Sustainable Chem. Eng. **11**(46), 16479–16490 (2023). <https://doi.org/10.1021/acssuschemeng.3c03918>
16. H. Luo, J. Liang, J. Zhou, Z. Yin, Z. Zhang et al., Synergistic coupling of FeOOH with Mo-incorporated NiCo LDH towards enhancing the oxygen evolution reaction. New J. Chem. **46**(17), 7999–8009 (2022). <https://doi.org/10.1039/d2nj00867j>
17. Y. Zhang, X. Song, S. Xue, Y. Liang, H. Jiang, Fabrication of hierarchically structured S-doped NiFe hydroxide/oxide electrodes for solar-assisted oxygen evolution reaction in seawater splitting. Appl. Catal. A Gen. **649**, 118965 (2023). <https://doi.org/10.1016/j.apcata.2022.118965>
18. L. Liu, Y. Chen, Q. Zhang, Z. Liu, K. Yue et al., Superhydrophilic NiFe-LDH@Co9S8-Ni3S2/NF heterostructures for high-current-density freshwater/seawater oxidation electrocatalysts. Appl. Catal. B Environ. Energy **354**, 124140 (2024). <https://doi.org/10.1016/j.apcatb.2024.124140>
19. Y. Chen, L. Dong, S. Jia, Q. Zhang, L. Liu et al., Superhydrophilic S-NiFe LDH by room temperature synthesis for enhanced alkaline water/seawater oxidation at large current densities. Small **21**(9), 2409499 (2025). <https://doi.org/10.1002/smll.202409499>
20. L. Wang, K. Huang, X. Zheng, Y. Liu, J. Wu et al., A corrosion-resistant amorphous/crystalline heterostructured catalyst for industrial-level seawater electrolysis in membrane electrode assembly electrolyzer. Adv. Funct. Mater. **35**(42), 2417603 (2025). <https://doi.org/10.1002/adfm.202417603>
21. H. Zhao, K. Deng, X. Liu, P. Liu, X. Lv et al., Promoted OH− adsorption and Cl− repulsion ability of Ce-decorated NiFe@CuO for alkaline seawater electrolysis. Adv. Funct. Mater. **35**(45), 2508539 (2025). <https://doi.org/10.1002/adfm.202508539>
22. L. Wu, L. Yu, B. McElhenny, X. Xing, D. Luo et al., Rational design of core-shell-structured CoP x @FeOOH for efficient seawater electrolysis. Appl. Catal. B Environ. **294**, 120256 (2021). <https://doi.org/10.1016/j.apcatb.2021.120256>
23. Y. Yang, W. Qiao, H. Yang, J. Li, Y. Huang et al., Corrosion engineering toward amorphous-crystalline nanoarray armored with tannin-nickel complex for industrial seawater electrolysis. Appl. Catal. B Environ. Energy **379**, 125669 (2025). <https://doi.org/10.1016/j.apcatb.2025.125669>
24. M. Qi, M. Qin, H. Wang, B. Lin, J. Chen et al., Regulating the redox cycle of nickel species for efficient seawater electrolysis. Appl. Catal. B Environ. Energy **356**, 124259 (2024). <https://doi.org/10.1016/j.apcatb.2024.124259>
25. S.A. Patil, A.C. Khot, V.D. Chavan, I. Rabani, D.-K. Kim et al., Electrostatically robust CoFeOF nanosheet against chloride for green-H2 production in alkaline seawater electrolysis. Chem. Eng. J. **480**, 146545 (2024). <https://doi.org/10.1016/j.cej.2023.146545>
26. P. Li, S. Zhao, Y. Huang, Q. Huang, B. Xi et al., Corrosion resistant multilayered electrode comprising Ni3N nanoarray overcoated with NiFe-phytate complex for boosted oxygen evolution in seawater electrolysis. Adv. Energy Mater. **14**(8), 2303360 (2024). <https://doi.org/10.1002/aenm.202303360>
27. L. Xiao, X. Bai, Z. Wang, C. Hou, J. Guan, Electron redistribution in Sn-NiFe oxyhydroxides for overall seawater electrolysis. Chem. Eng. J. **521**, 166872 (2025). <https://doi.org/10.1016/j.cej.2025.166872>
